# Seasonal variation modulates coral sensibility to heat-stress and explains annual changes in coral productivity

**DOI:** 10.1038/s41598-017-04927-8

**Published:** 2017-07-10

**Authors:** Tim Scheufen, Wiebke E. Krämer, Roberto Iglesias-Prieto, Susana Enríquez

**Affiliations:** 1 Laboratorio de Fotobiología. Unidad Académica de Sistemas Arrecifales Puerto Morelos, Universidad Nacional Autónoma de México, (UNAM), Prolongación Avenida Niños Héroes S/N, domicilio conocido, Puerto Morelos, Cancún, Q.R. 77580 Mexico; 2 Posgrado de Ciencias del Mar y Limnología (PCMyL) of the Universidad Nacional Autónoma de México (UNAM), Mexico City, Mexico; 3 Department of Biology, The Pennsylvania State University, 208 Mueller Laboratory, University Park, PA 16802 USA

## Abstract

The potential effects of seasonal acclimatization on coral sensitivity to heat-stress, has received limited attention despite differing bleaching thresholds for summer and winter. In this study, we examined the response of two contrasting phenotypes, termed winter and summer, of four Caribbean reef corals to similar light and heat-stress levels. The four species investigated were categorized into two groups: species with the ability to harbour large number of symbionts, *Orbicella annularis* and *O*. *faveolata*, and species with reduced symbiont density (*Montastraea cavernosa* and *Pseudodiploria strigosa*). The first group showed higher capacity to enhance photosynthetic rates per area (P_max_), while P_max_ enhancement in the second group was more dependent on *Symbiodinium* performance (P_sym_). In summer all four species presented higher productivity, but also higher sensitivity to lose coral photosynthesis under heat-stress. In contrast, corals in winter exhibit symbionts with higher capacity to photoacclimate to the increased levels of light-stress elicited by heat-stress. Overall, our study supports the importance of the acclimatory coral condition in addition to the previous thermal history, to determine the severity of the impact of heat-stress on coral physiology, but also the dependence of this response on the particular structural and functional traits of the species.

## Introduction

Scleractinian corals form ecologically obligate symbioses with photosynthetic dinoflagellates in the genus *Symbiodinium*, which are fundamental for the construction and maintenance of the primary framework of coral reefs. For some species, up to 90% of the organic carbon fixed by the symbionts through photosynthesis is translocated to the coral host^1^ to support coral calcification^2^. The symbioses between coral hosts and *Symbiodinium* are highly dynamic, showing seasonal variations in pigmentation, protein content, and symbiont density^3–6^, as well as in photosynthesis^7–9^ and calcification^10, 11^. Seasonal changes in coral pigmentation and symbiont density usually shift in opposite phase relative to calcification, as the warmer season often presents lower symbiont number and pigmentation^5, 6^ with the highest calcification^10^. Therefore, corals may be differentiated by two seasonally driven phenotypes: lower pigmented summer holobionts (host-symbiont unit), which seem to be more productive with respect to their ability to calcify, and more pigmented but less productive winter holobionts.

Seasonal changes in temperature and light are the main drivers of seasonal variation in coral pigmentation^4^. The combined effect of temperature and light has also been recognized as the main trigger of severe losses in coral pigmentation, known as coral bleaching^12, 13^, which commonly involve large symbiont declines and minimal loss of pigment per symbiont. Coral bleaching is induced by prolonged exposure to water temperatures of +1 °C above the local maximum of the (summer) monthly mean (MMM) sea surface temperature (SST), which results in a strong functional perturbation of the symbiotic association^14^. Accordingly, coral bleaching culminates not only in severe losses of coral pigmentation and symbionts, but also in a dysfunctional holobiont. When corals do survive the elevated temperature event and the stress has ceased, the symbiotic association can be re-established and the physiology of the holobiont may recover^15, 16^.

Susceptibility to bleaching varies largely among species^17, 18^ but has been shown to be related to intrinsic coral characteristics, such as colony morphology^18^, tissue thickness^18, 19^, the photosynthetic physiology of the dominant *Symbiodinium* type harboured^20, 21^, overall energy level of the organism^22^ and/or holobiont capacity to cope with the high level of oxidative stress induced by heat-stress within coral tissues^23^. In addition to the wide array of possible factors influencing the sensitivity of symbiotic corals to heat- and light-stress, holobiont seasonal acclimatization may play an important role in the regulation of these responses. This interpretation is supported by the documented differences in bleaching thresholds between summer and winter^24^, although little attention has been given to this issue. In contrast, significant scientific effort is currently focused on understanding the capacity of coral adaptation to global warming and ocean acidification, whereas the role of seasonal acclimatization is not yet fully understood and often overlooked. To understand the ecological role of coral acclimatization in relation to coral susceptibility to elevated temperature, we exposed corals that had already developed the complete expression of two holobiont phenotypes at the end of winter and end of summer, to similar light and heat-stress levels. The Caribbean coral reef builders characterized were: *Orbicella annularis*, *O*. *faveolata*, *Montastraea cavernosa*, and *Pseudodiploria strigosa*. Two experiments were performed, one in March 2011, at the end of the cold season, and the other in October 2011, end of the warm season, when the organisms were acclimated to comparable water temperature, near the annual mean for the Puerto Morelos reef lagoon (Mexican Caribbean, local annual SST average ≈ 28 °C)^25^. We exposed experimental organisms for 10 days to control (28 °C), moderate (+2 °C = 30 °C) and severe (+4 °C = 32 °C) temperature regimes in outdoor mesocosm facilities at UNAM (UASA-UNAM, Puerto Morelos, Mexico). Before experimentation, we examined the response of each species and holobiont condition to short-term incubations, at five temperatures, which ranged from 26 °C to 34 °C. The two experiments were performed using natural solar radiation and under two contrasting transitional seasons due to the Sun’s declination. This allowed for analysis of the Sun’s potential direct effect on holobiont physiology. In addition to small and progressive changes in diurnal irradiance, significant differences in cloud cover were also present between the two seasons^25^. To solve this problem, we used three irradiance levels (high-HL, medium-ML and low-LL) and randomly shuffled the organisms among the three light treatments in order to generate similar highly-variable experimental light regimes between both experiments. These daily fluctuations in light exposure simulated the passing of storm events, which rarely occurred in March, but are frequent enough in September-October on the coast of the Mexican Caribbean.

## Results

**Figure 1:**
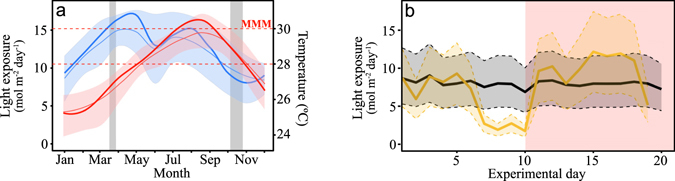
Description of the natural and experimental variation in light and temperature. (**a**) Monthly average values (thin lines) ± STD (shaded areas) for the annual variation of diurnal light exposure (mol quanta m^−2^ day^−1^) for the period 2011 to 2014 (in blue), and seawater temperature (°C) for the period 1992 to 2015 (in red). Thick solid lines describe average values during 2011for light exposure (blue line) and temperature (red line). Dashed horizontal red lines indicate annual average seawater temperature (28 °C), and local value for MMM (29.8 ≈ 30 °C), for the reef lagoon of Puerto Morelos. (**b**) Diurnal variation in light exposure (mol quanta m^−2^ day^−1^) during the experiments, values for March 2011 in grey, and for October 2011 in orange. The solid line represents diurnal light exposure for the medium-control light treatment (ML), whereas the upper and lower dashed lines describe, respectively, high light (HL) and low light (LL) treatment values. The red area from day 10 to day 20 shows the variation in diurnal light exposure during the extended 10 days of heat-stress applied in October 2011. In March, corals were back to control conditions (28 °C) after the application of the heat-stress treatments for 10 days.

Light exposure for the 2011–2014 year period showed two annual peaks (May and July) and significant inter-annual variation. A progressive increase in diurnal irradiance was observed from January to May, with a decline for the period between July and December (Fig. 1a). In 2011, the second peak in light exposure was observed in August followed by a faster decline (Fig. 1a). Seawater temperature showed a delayed seasonal fluctuation with respect to light exposure, with a single peak in August (Fig. 1a). In 2011 we observed a faster increase in seawater temperature, compared with the average value of the period 1992–2015, and higher maxima with some periods in August above the local MMM of ~30 °C. During coral sampling and *in situ* recovery in the reef lagoon, corals were exposed, on average, to 26.2 °C (±0.5 °C, March) and 29.9 °C (±0.8 °C, October). Larger differences were observed between both seasons in the previous thermal regimes. In January 2011 corals had been exposed to minimum values of 25.2 °C, while in August–September 2011 they had experienced an average maximum of 30.5 °C, with daily peaks of up to 31 °C. The short periods of seawater temperature above the local MMM of ~30 °C that occurred in the summer of 2011, were not long enough to be registered by the NOAA DHW (*Degree Heating Weeks*) algorithm^26^ (see http://www.ospo.noaa.gov/Products/ocean/cb/dhw/2011.html).

To investigate the effect of coral acclimatization and previous thermal-regime on coral responses to heat stress, we characterized each seasonal holobiont condition before performing the experimental analysis. Two contrasting holobionts were described, termed “winter phenotype” (March) and “summer phenotype” (October).

### Differences between winter and summer phenotypes and among coral species

**Figure 2:**
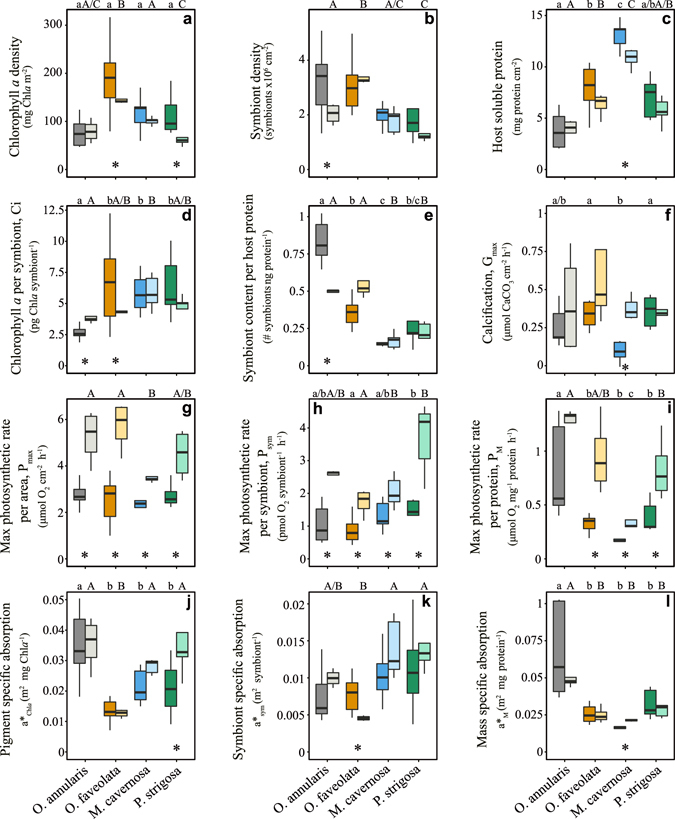
Characterization of winter and summer coral phenotypes. Box plots describing the variability of the different descriptors used in this study to characterize both winter (dark color) and summer (light color) phenotypes of *Orbicella annularis* (grey), *Orbicella faveolata* (orange), *Montastraea cavernosa* (blue), and *Pseudodiploria strigosa* (green). Boxes encompass the 25 and 75% quartiles of all the data. The central line corresponds to the median, and bars extend to the 95% and 5% of the confidence limits. Asterisks indicate significant differences (t-test, p < 0.05) between phenotypes within species.

Significant changes between phenotypes and among species were observed for all structural and functional coral traits investigated (Fig. 2, Tables S1–S3 in supplementary material). Chlorophyll *a* (Chl*a*) and soluble host protein normalized to area were generally lower in the summer phenotypes of all species except for the low-pigmented *O*. *annularis*, which also showed the lowest host protein content (Fig. 2a,c). Non-significant seasonal changes in Chl*a* were also found for *M*. *cavernosa*, which was the only species that presented in summer significant declines in soluble host proteins (Fig. 2b,c). The lack of a change in pigmentation found for *O*. *annularis* was masked by significant declines in symbiont density (38%) concomitant with increases in chlorophyll *a* per symbiont (Ci, 50%, Fig. 2b,d). On the other hand, the summer reductions in holobiont pigmentation of *P*. *strigosa* and *O*. *faveolata* showed a contrasting pattern, as *P*. *strigosa* experienced large declines in symbiont number (44%), while the reduction in *O*. *faveolata* pigmentation was due to Ci declines as slight increases in the number of symbionts were also found (Fig. 2a,b,d; Tables S1–S2). Changes in symbiont number for both species and also the reductions estimated for *M*. *cavernosa* were not significant (Fig. 2b; Tables S1–S2).

Coral calcification increased in summer significantly for *M*. *cavernosa* (409%), but the higher rates estimated for *O*. *annularis* (57%) and *O*. *faveolata* (207%) were not statistically significant. *P*. *strigosa* showed no change between seasons (Fig. 2f, Tables S1–S2). Photosynthetic rates per area (P_max_; µmol O_2_ cm^−2^ h^−1^), per protein content (P_M_; µmol O_2_ protein^−1^ h^−1^), and per symbiont (P_sym_; pmol O_2_ sym^−1^ h^−1^) were higher in the summer phenotype of all species, except for the P_M_ of *O*. *annularis* (Fig. 2g–i). In summer both *Orbicella spp* presented the highest rates (Fig. 2g–i, Tables S1–S2), which were doubled in *O*. *faveolata* with respect to the average winter rates (Fig. 2g). The highest P_M_ was achieved by *O*. *annularis*, while *P*. *strigosa* presented the highest P_sym_ (Fig. 2g–i). In contrast, *M*. *cavernosa* showed in summer the lowest P_max_ and P_M_ (Fig. 2g,i), and *O*. *faveolata* the lowest P_sym_ (Fig. 2h). In winter, we did not detect differences among species in P_max_ but significant changes were found between *O*. *faveolata* and *P*. *strigosa* for P_sym_, and between *O*. *annularis* and the other three species for P_M_ (Tables S1 and S2). No significant changes between phenotypes and among species in post-illumination coral respiration were observed (Tables S1–S2).

With respect to the optical traits, we used in this study two new optical descriptors in addition to the previously characterized pigment specific absorption (a*_Chl*a*_; m^2^ mg Chl*a* ^−1^; see Enríquez *et al*. 2005): the specific absorption coefficients normalized to symbiont number (a*_sym_; m^2^ sym^−1^; Fig. 2j,k; Tables S1–S2) and to soluble host protein content (a*_M_; cm^2^ mg protein^−1^). The interest of these parameters was to characterize, respectively, symbiont-specific and host mass-specific light absorption efficiencies^27, 28^. All species, excluding *O*. *faveolata*, developed in summer more efficient holobionts (a*_Chl*a*_) and symbionts (a*_sym_) for collecting solar energy (Fig. 2j,k; Tables S1–S2). However, host mass-specific efficiency (a*_M_) did not present any clear seasonal pattern (Fig. 2i), except for the thickest species *M*. *cavernosa* (Fig. 2l; Tables S1–S2), which also showed in summer higher efficiency. *P*. *strigosa* and *O*. *annularis* were in summer the most efficient light collectors (a*_Chl*a*_; Fig. 2j), while *P*. *strigosa* and *M*. *cavernosa* harboured the most efficient symbionts to collect light in both seasons (a*_sym_; Fig. 2k).

**Figure 3:**
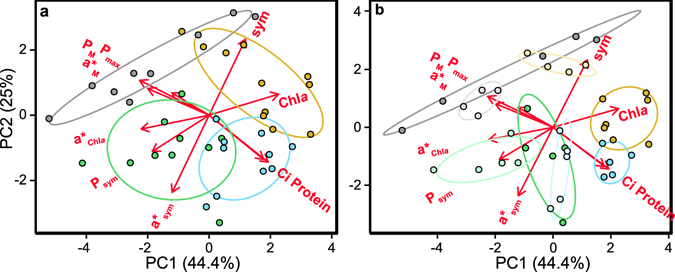
Principal component analysis (PCA) for the four coral species and two phenotypes investigated﻿:. (**a**) grouping of the control specimens by species: *O*. *annularis* (grey), *O*. *faveolata* (orange), *M*. *cavernosa* (blue), and *P*. *strigosa* (green), based on the variability in their structural (Chl*a*, symbiont, Ci, protein content), optical (a*_Chl*a*_, a*_sym_, a*_M_) and photo-physiological (P_max_, P_sym_, P_M_) coral traits. Red arrows indicate the correlation of the different descriptors with PC1 and PC2; and (**b**) same analysis splitting each species by phenotype (summer phenotype in a lighter color).

Principal component analysis (PCA) highlighted the structural and functional variability better related with the differences found among the four coral species and the two seasonal phenotypes characterized (Fig. 3a). The first principal component (PC1), which described 44% of the variance, was primarily determined by structural changes in Chl*a*, symbiont and soluble host protein content, negatively associated with functional changes in a*_Chl*a*_, a*_M_, P_M_ and P_sym_ (Fig. 3; Table 1). The second principal component (PC2), which enhanced the variance explained to 69%, was mainly described by symbiont density, negatively associated with Ci, soluble host protein and a*sym (Fig. 3; Table 1). Finally, the third component (PC3) allowed increasing the variability explained to 85%, thanks to the contribution of two optical descriptors, a*_Chl*a*_ and a*_M_, negatively associated with P_max_ and P_sym_ (Fig. 3; Table 1). According to these results, the highly pigmented corals, *O*. *faveolata* and *M*. *cavernosa*, showed the lowest values for the optical descriptors, in particular a*_Chl*a*_ and a*_M_, but also achieved such high pigmentation through two contrasting strategies: increasing the number of symbionts (*O*. *faveolata*) or increasing *Symbiodinium* Ci (*M*. *cavernosa*). These two species showed similar holobionts in winter, but presented large differences in the summer phenotypes (Fig. 3b). In contrast, *P*. *strigosa* and *O*. *annularis* may represent two opposite strategies or “evolutionary solutions” relative to *O*. *faveolata* and *M*. *cavernosa*. *P*. *strigosa* presented higher a*_Chl*a*_, a*_sym_ and P_sym_ than *O*. *faveolata*, especially in summer (Fig. 3b), while *O*. *annularis* showed higher values for a*_M_ but also for P_M_ and P_max_, relative to *M*. *cavernosa* (Fig. 3b). As mentioned, the smallest differences between seasonal phenotypes were found for *O*. *annularis*.

**Table 1:** Principal component analysis (PCA) loadings for the variation of the structural, optical, and photo-physiological coral traits of the winter and summer coral phenotypes (control organisms).

	PC1	PC2	PC3
Chlorophyll *a* (mg Chl*a* m^−2^)	**0**.**39**	0.15	−0.10
Symbionts (x10^6^ # sym cm^−2^)	0.20	**0**.**53**	0.02
Ci (mg Chl*a* sym^−1^)	**0**.**31**	**−0**.**31**	−0.11
Soluble Host Protein (mg protein cm^−2^)	**0**.**33**	**−0**.**33**	−0.10
a*_chl*a*_ (m^2^ mg Chl*a* ^−1^)	**−0**.**38**	−0.10	**0**.**32**
a*_sym_ (m^2^ sym^−1^)	−0.21	**−0**.**54**	0.10
a*_M_ (m^2^ mg protein^−1^)	**−0**.**34**	0.19	**0**.**38**
P_max_ (µmol O_2_ cm^−2^ h^−1^)	−0.21	0.16	**−0**.**67**
P_sym_ (pmol O_2_ sym^−1^ h^−1^)	**−0**.**32**	−0.26	**−0**.**45**
P_M_ (µmol O_2_ protein^−1^ h^−1^)	**−0**.**39**	0.25	−0.24
Standard deviation	2.11	1.58	1.26
Proportion of Variance	0.44	0.25	0.16
Cumulative Proportion	0.44	0.69	0.85

The cumulative variation accounted for by each principal component is also shown.

In bold are highlighted the highest correlations [loading > 0.3] between parameters and PCs.

### Scaling quotient of temperature (Q_10_)

**Figure 4:**
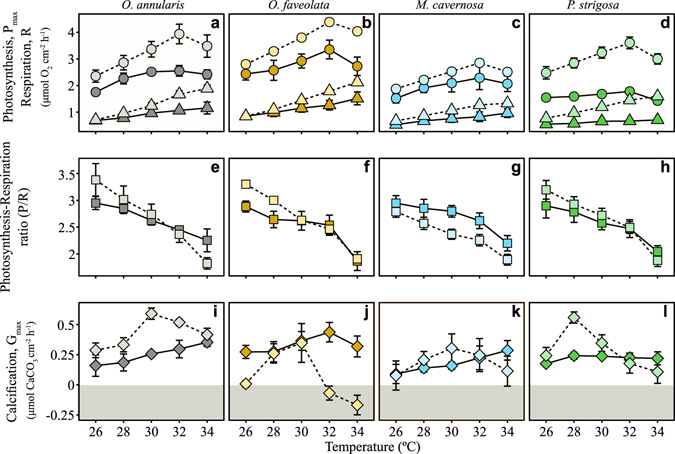
Scaling quotient of temperature (Q_10_). Mean ± SE (n = 5) of the maximum gross photosynthesis rates (P_max_, circles), post-illumination respiration rates (triangles), P/R ratio (squares), and calcification rates (diamonds), for the winter (dark color) and summer (light color) phenotypes of *Orbicella annularis* (grey), *Orbicella faveolata* (orange), *Montastraea cavernosa* (blue), and *Pseudodiploria strigosa* (green). Negative values for coral calcification (grey area) are considered as indication of coral decalcification activity.

Temperature enhanced all metabolic rates along an optimal range, also negatively affecting some processes when increased above a particular threshold (Fig. 4). Adverse effects on coral photosynthesis were observed for all species and phenotypes above 32 °C, but no negative impact was detected for coral respiration over the range of temperature investigated (Fig. 4a–d). Coral photosynthesis and respiration showed higher temperature scaling factors, Q_10_, in the summer phenotypes (Tables S3–S4), which resulted in stronger declines in holobiont P/R ratios (Fig. 4e–h). However, this descriptor was maintained above 1 for all species and phenotypes in the short incubations employed in this study, even at the highest temperature analyzed (Fig. 4e–h). The largest differences among phenotypes were found in coral calcification (Fig. 4i–l; Tables S3–S4). In winter, only *O*. *faveolata* presented an adverse effect of temperature above 32 °C. In contrast, all species showed adverse effect in summer, either above 28 °C (*P*. *strigosa*, Fig. 4l) or above 30 °C (the two *Orbicella spp*. and *M*. *cavernosa*, Fig. 4i–k). For *O*. *faveolata* negative values (i.e. decalcification rates) were estimated above 32 °C.

### Coral responses to thermal-stress

Experimental organisms were exposed in March and October 2011 to 28 °C (control) and +2 °C and +4 °C heat-stress treatments. For all species analysed, the severe temperature treatment led to most dramatic structural and functional modifications.

### Structural changes

**Figure 5:**
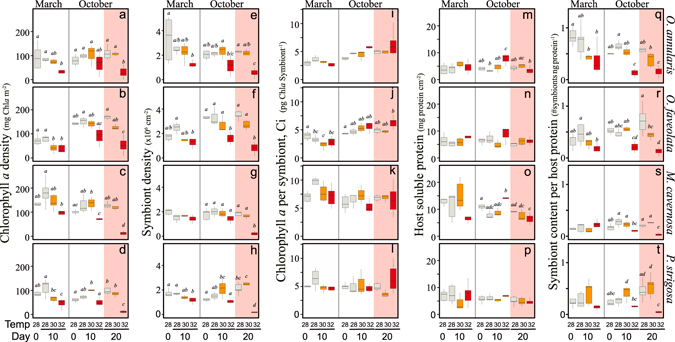
Response of coral structural descriptors to heat-stress. Box plots describing the variability of the structural descriptors for the four coral species investigated: *Orbicella annularis*, *Orbicella faveolata*, *Montastraea cavernosa*, and *Pseudodiploria strigosa*. Each plot describes coral responses in March (winter phenotype) and October (summer phenotype) after 10 days (white area) and 20 days (red area) of exposure to control (28 °C; grey), and heat-stress conditions of +2 °C (30 °C; orange), and +4 °C (32 °C; red). Boxes encompass the 25 and 75% quartiles of all the data (n = 5). The central line corresponds to the median, and bars extend to the 95% and 5% of confidence limits. Letters mark significant differences (Tukey Post-Hoc test, p < 0.05) between treatments within a season.

In March 2011 Chl*a* density was significantly reduced at 32 °C in all species (Fig. 5a–d; Tables S6–S7), with *O*. *annularis* showing the largest decline (65%) and *M*. *cavernosa* the smallest (26%). However, in October 2011, the reduction in pigmentation after 10 experimental days at 32 °C was not significant for any species (Tables S8–S9). Prolonged exposure to 32 °C (10 more days) caused large reductions in Chl*a* in all species, with *M*. *cavernosa* and *P strigosa* loosing ~80% of their initial values (Fig. 5a–d). Symbiont density declined significantly in March 2011 in *O*. *annularis* (62%), *O*. *faveolata* (53%), and *P*. *strigosa* (31%) after 10 days at 32 °C (Fig. 5e–h; Tables S6–S7). Large reductions in symbiont density were also observed in October 2011 for the two *Orbicella spp*., but not for *M*. *cavernosa* and *P*. *strigosa*. The extended heat stress further caused significant decreases of symbiont density in all species, with the largest decline observed for *M*. *cavernosa* and *P*. *strigosa* (~80%, Fig. 5g,h; Tables S8–S9).

Chlorophyll *a* per symbiont (Ci) was highly variable among replicates, which resulted in a lack of significance for most comparisons (Fig. 5i–l; Tables S6–S9). In March 2011, significant Ci reductions were only observed in *O*. *faveolata* after 10 days at 30 °C (Tables S6–S7). This treatment induced for this species larger declines in *Symbiodinium* Ci than exposure to 32 °C. In March, *Symbiodinium* tended to increase Ci under control conditions in *M*. *cavernosa*, *P*. *strigosa* and *O*. *annularis*. However, under elevated temperature Ci values were progressively reduced (Fig. 5i–l), although changes were only significant for *M*. *cavernosa* (Tables S6–S7). In October 2011, we observed the opposite response for the two *Orbicella spp*., with increases in Ci after exposure to heat-stress (Fig. 5i–l). The largest increases were estimated for the symbionts of *O*. *annularis* exposed to 32 °C (49%), which showed further increases in its Ci during the extended stress (Fig. 4j).

*M*. *cavernosa* was the only species to reduce soluble host protein at 32 °C in March 2011 (Fig. 5o, Tables S6–S7). In October 2011, host protein values were less variable among replicates and increased for all species at 32 °C, although changes were only significant for *O*. *annularis* and *M*. *cavernosa* (Fig. 5m–p; Tables S8–S9). The extended heat stress resulted in reductions in soluble host protein for all species, except for *O*. *faveolata* (Fig. 5m–p), although changes were only significant for *M*. *cavernosa* (Tables S8–S9). Finally, the number of symbionts normalized to protein content decreased in March 2011 with exposure to heat-stress except in *M*. *cavernosa* (Fig. 5q–t). These reductions were strongest for the two *Orbicella spp* in both experiments, which declined at 32 °C between 39% and 76% (Fig. 5q–t). *M*. *cavernosa* and *P*. *strigosa* also showed in October reduced values for symbiont number normalized to protein content.

### Maximum photochemical efficiency of photosystem II (F_v_/F_m_)

**Figure 6:**
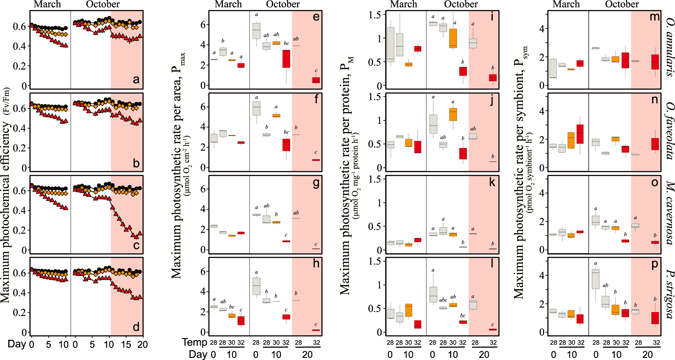
Response of coral photosynthesis to heat-stress. (**a**–**d**) Daily averages for *F*_*v*_ */F*_*m*_ recorded at dusk (n > 10) for corals exposed to control (28 °C, black triangles), 30 °C (orange triangles) and 32 °C (red triangles) in March (winter phenotype) and October (summer phenotype). (**e–p**) Box plots describing the variability of the photosynthetic responses of the four coral species investigated: *Orbicella annularis*, *Orbicella faveolata*, *Montastraea cavernosa*, and *Pseudodiploria strigosa*. Each plot describes coral responses in March (winter phenotype) and October (summer phenotype), after 10 days (white area) and 20 days (red area) of exposure to control (28 °C; grey), 30 °C (orange), and 32 °C (red). Boxes encompass the 25 and 75% quartiles of all the data (n = 5). The central line corresponds to the median, and bars extend to the 95% and 5% of the confidence limits. Letters mark the significant differences (Tukey Post-Hoc test, p < 0.05) between treatments within a season.

This parameter presented in March 2011 progressive daily reductions throughout the 10 days experiment. Such decline was proportional to the heat-stress treatment: moderate at 30 °C and large at 32 °C (Fig. 6a–d). A significant small decline was also observed for control (28 °C) specimens of *M*. *cavernosa* and *P*. *strigosa* (Fig. 6c,d; Tables S10–S11). In October 2011, the progressive *F*_*v*_ */F*_*m*_ reduction was only observed until day 6, followed by recovery or maintenance of similar *F*_*v*_ */F*_*m*_ values until day 10 (Fig. 6a–d; Tables S10–S11). This recovery was associated with the overcast conditions produced by Hurricane Rina, which dramatically reduced diurnal irradiance after day 6. Light levels were back to initials or even higher after day 11 (Fig. 1b). This implies that the experimental corals during the extended 10 experimental days were exposed to higher irradiances (Fig. 1b). However, control and 30 °C organisms did not experience significant changes in *F*_*v*_ */F*_*m*_, while at 32 °C all species accumulated significant photodamage (photosystem II, PSII, inactivation) (Fig. 6a–d). Large *F*_*v*_ */F*_*m*_ reductions were observed in *M*. *cavernosa* (73%) and *P*. *strigosa* (40%), and moderate in *O*. *faveolata* (27%), and *O*. *annularis* (21%; Fig. 6a–d). The strongest impact on *F*_*v*_ */F*_*m*_ was observed in March for nubbins of *M*. *cavernosa* and *O*. *annularis* exposed to 32 °C (34–35% reduction) (Fig. 6a,c), but all species and treatments, except specimens of *O*. *faveolata* exposed to control and 30 °C, experienced significant changes in *F*_*v*_ */F*_*m*_ at day 10 (Tables S10–S11). In contrast, in October 2011 only the 32 °C treatment induced a significant impact on *F*_*v*_ */F*_*m*_ (Fig. 6a–d).

### Variation of holobiont photosynthesis per-area-P_max_ and per-protein-P_M_

In March 2011, *P*. *strigosa* was the only species that showed a significant reduction in P_max_ at elevated temperature (Fig. 6h; Tables S6–S7) despite the large changes in pigmentation observed in all species. The two *Orbicella spp*. increased P_max_ after 10 days at 28 °C, although only *O*. *annularis* showed significant changes (Fig. 6e–h; Tables S6–S7). In October, however, all species experienced significant P_max_ reductions, specially at 32 °C (Fig. 6e–h; Tables S8–S9). Prolonged heat-stress resulted in a full suppression of P_max_ in the specimens of *M*. *cavernosa* and *P*. *strigosa* exposed to 32 °C, and in severe declines for those of *O*. *annularis* (90%) and *O*. *faveolata* (87%).

Holobiont photosynthesis per protein (*P*_*M*_) showed after 10 days at 32 °C in March small reductions in the two *Orbicella spp*. and *P*. *strigosa*, although non-significant, whereas no decline was observed in *M*. *cavernosa* (Fig. 6i–l; Tables S8–S9). In October, however, all species significantly decreased their *P*_*M*_ at this temperature (Fig. 6i–l, Tables S8–S9).

### Variation of the contribution of Symbiodinium to holobiont photosynthesis, P_sym_

No significant changes were observed in P_sym_ in March 2011 for any species or treatment (Fig. 6m–p; Tables S6–S7). However, in October 2011, a negative trend was evident for *P*. *strigosa* and *M*. *cavernosa*, after exposure to heat-stress (Fig. 6o,p; Tables S8–S9). In the two *Orbicella spp*., P_*sym*_ did not change significantly among treatments (Fig. 6m,n; Tables S8–S9). The extended heat-stress (day 20) showed similar P_*sym*_ to the initial values in the two *Orbicella spp*., while in *M*. *cavernosa* and *P*. *strigosa* they were similarly reduced to the values determined at day 10 (Fig. 6o,p; Tables S8–S9).

**Figure 7:**
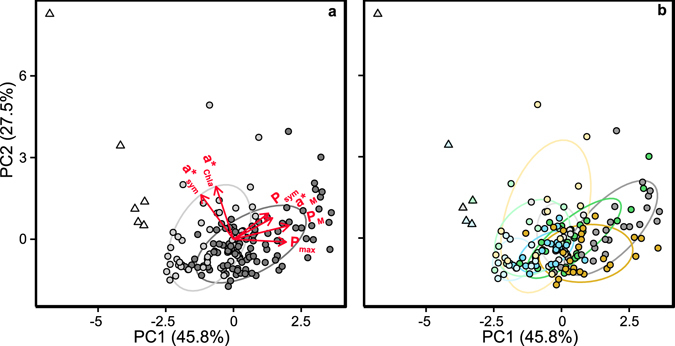
Principal component analysis (PCA) for control and heat-stressed corals. (**a**) grouping of control (dark grey circles), heat-stressed (grey circles) and bleached (light grey triangles) coral phenotypes, based on optical (a*_Chl*a*_, a*_sym_, a*_M_) and photo-physiological (P_max_, P_sym_, P_M_) coral traits. Red arrows indicate the correlation of the different descriptors with PC1 and PC2. (**b**) grouping of control, heat-stressed and bleached corals based on the same descriptors per species: *O*. *annularis* (blue), *O*. *faveolata* (orange), *M*. *cavernosa* (green), and *P*. *strigosa* (red). Different shading illustrates differences from unstressed (dark) to bleached (lighter) phenotypes.

Principal component analysis (PCA) highlighted the functional parameters that better described the variability induced by heat-stress (Fig. 7). The first principal component (PC1), which described 46% of the variance, was primarily determined by the photosynthetic parameters, P_M_, P_max_, and even P_sym_, negatively associated with a*_sym_ but positively associated with a*_M_ (Fig. 7, Table 2). The second principal component (PC2) increased the variability explained to 73% and was mainly determined by a*_Chl*a*_ and a*_sym_ although P_sym_ also presented a significant contribution (Fig. 7, Table 2). With the third component (PC3) the variability described rose to 90% (Table 2), thanks to the contribution of a*_M_, negatively associated with P_sym_ and positively with P_max_ (Table 2).

**Table 2:** Principal component analysis (PCA) loadings for the variation of the optical and photo-physiological traits of control and heat-stressed corals.

	PC1	PC2	PC3
a*_chl*a*_ (m^2^ mg Chl*a* ^−1^)	−0.17	**0**.**68**	−0.06
a*_sym_ (m^2^ sym^−1^)	**−0**.**32**	**0**.**57**	0.11
a*_M_ (m^2^ mg protein^−1^)	**0**.**39**	0.27	**−0**.**65**
P_max_ (µmol O_2_ cm^−2^ h^−1^)	**0**.**53**	−0.03	**0**.**35**
P_sym_ (pmol O_2_ sym^−1^ h^−1^)	**0**.**35**	**0**.**33**	**0**.**62**
P_M_ (µmol O_2_ protein^−1^ h^−1^)	**0**.**56**	0.18	−0.22
Standard deviation	1.66	1.29	0.99
Proportion of Variance	0.46	0.28	0.16
Cumulative Proportion	0.46	0.73	0.90

The cumulative variation accounted for by each principal component is also shown.

In bold are highlighted the highest correlations [loading > 0.3] between parameters and PCs.

## Discussion

In this comparative study we observed, as expected, contrasting responses among coral species to similar levels of heat-stress. However, we also found significant differences between winter and summer coral phenotypes for the four species investigated. Interestingly, our results also revealed large similarities among species in the seasonal acclimatization of the holobiont, and in the susceptibility of each seasonal phenotype to light- and heat-stress. The structural and physiological characteristics of each phenotype agrees with previous seasonal descriptions^5, 6, 19^, and supports the association between seasonal holobiont adjustments^3–6, 29^ and the annual variability documented for coral performance^7–11, 29^. The summer phenotype showed higher calcification and photosynthesis rates per area, per symbiont and per host-mass, consistent with the widely documented higher summer growth rates^10^ and calcium carbonate production^7–9, 11^ of symbiotic scleractinian corals. The winter type also presented no adverse impact of elevated temperature on the calcification rates of three species, and lower temperature scaling quotients for all species investigated (Q_10_). The only species showing an adverse response to elevated temperature in the winter type, *O*. *faveolata*, also suffered the largest declines in coral calcification when exposing the summer phenotype to heat-stress. In contrast, this species displayed the most robust response of holobiont photosynthesis to heat-stress in both phenotypes. Overall, the characterization of the scaling quotients (Q_10_) revealed significant reductions in coral calcification after exposure to elevated temperature for the four species investigated, larger for the summer phenotypes of all coral species. This implies that an inherent seasonal component in the holobiont in addition to the external seasonal fluctuation of the environment, may control annual calcium carbonate production. Such inherent component also affects coral sensitivity to heat-stress and could explain the different coral bleaching thresholds documented in summer and winter^24^.

Understanding the metabolic/cellular processes behind holobiont seasonal adjustments is beyond the scope of this study. However, the wide repertoire of quantitative coral morpho-functional traits analysed, can aid to develop a new framework for the recently proposed “*Trait*-*Based approach”* in coral reef research^30^. In addition to the traditional morpho/structural, demographic coral descriptions, or the proposed incorporation of plant traits derived from plant ecology^30^, the development of quantitative functional descriptors^28, 31–33^ to parameterize key physiological processes of these symbioses, such as organic and inorganic carbon production or resource acquisition efficiency, can be more useful for the development of the “*Coral Trait*-*Based”* approach. In this context, the four species investigated can be categorized into two groups: species with the ability to harbour large number of symbionts and that display a highly variable symbiont density, such as *O*. *annularis* and *O*. *faveolata*; and species with reduced symbiont density and also variability in this coral trait (*M*. *cavernosa* and *P*. *strigosa*). The first group showed in our comparison higher capacity to enhance photosynthetic rates per area, P_max_, particularly in the summer phenotype, while P_max_ enhancement was in the second group more dependent on *Symbiodinium* performance (P_sym_). *O*. *faveolata* and *P*. *strigosa* would represent two contrasting strategies for the optimization of the contribution of *Symbiodinium* to holobiont photosynthesis (P_sym_), as reflected in the PCA analysis. According to this analysis (Fig. 3), but also to the characteristics of the summer phenotypes reflected in Fig. 2, *P*. *strigosa* was able to achieve similar photosynthetic rates per area to *O*. *annularis* at much lower symbiont density, thanks to a significant enhancement in *Symbiodinium* photosynthesis (P_sym_, Fig. 2h). Such optimization was also associated according to the PCA analysis, with higher light absorption efficiency (a*_sym_). Changes in the genetic identity of the dominant *Symbiodinium* type may also respond to holobiont optimization of photosynthesis and calcification rates^34–36^, although host ability to enhance *Symbiodinium* performance through the regulation of host-dependent resources such as light^31, 37^, carbon and/or nitrogen supply^22^ cannot be neglected. PCA analysis also highlighted the relevance of soluble host protein content and *Symbiodinium* pigmentation (Ci) to characterize significant differences among species. The species that showed the lowest values for both partners of this symbiosis, *O*. *annularis*, presented the highest holobiont photosynthetic production and the highest light absorption efficiency per host mass (a*_M_). In contrast, *M*. *cavernosa* would represent the opposite evolutionary solution (lowest a*_M_ and P_M_), showing in this comparison the lowest photosynthesis performance in summer and the lowest calcification performance in winter (Fig. 2).

Exposure to heat-stress resulted for the four coral species and the two phenotypes characterized in significant *in hospite* accumulation of photodamage in *Symbiodinium*. When comparing the first 10 experimental days, larger accumulation was observed in the winter phenotype, irrespective of the species and the potential variability in the dominant *Symbiodinium* type, unfortunately not accounted in this study. The only species that did not show this response, *P*. *strigosa*, presented the most robust algal response to light-stress *in hospite*, except for the extended heat-stress performed in October (Fig. 6). Our comparison, thus, agrees with previous studies^38–42^, which documented that *Symbiodinium* light-stress *in hospite* is exacerbated by elevated temperature. Our results also support the specificity of this response^43^, as *M*. *cavernosa* and *O*. *annularis* showed the strongest impact and largest losses of symbionts, while *O*. *faveolata* and *P*. *strigosa* presented the most robust responses. A similar and species-specific enhancement of light-stress after exposure to heat-stress has been recently reported for coralline algae^44^. We also document for the first time in this study that the severity of the impact of heat-stress on *Symbiodinium in hospite*, measured as *F*_*v*_ */F*_*m*_ declines, has an inherent component in addition to the effect of seasonal fluctuations in light and temperature on *F*_*v*_ */F*_*m*_ previously documented^29^, which has to be related to the holobiont seasonal condition. Surprisingly, the largest *F*_*v*_ */F*_*m*_ declines observed for the winter phenotype were not expressed in similar reductions in coral photosynthetic rates. On the contrary, coral photosynthesis was less affected by heat-stress in all winter phenotypes. This observation is a call of attention to the assimilation of *F*_*v*_ */F*_*m*_ declines to photoinhibition and, thus, reductions in coral photosynthesis. *F*_*v*_ */F*_*m*_ declines reflect the initial step of the acclimatory response of any photosynthetic organism to increasing high-light stress. Thus, the absence of photosynthesis photoinhibition in the winter phenotype reflects the occurrence of a more efficient high-light photoacclimatory response of *Symbiodinium in hospite*. This high-light response is also supported by the large declines in *Symbiodinium* Ci (Fig. 5) found in March, which resulted in large losses in coral pigmentation (i.e, chlorophyll *a* density) but lower changes in symbiont number. This response was not observed in October, when we estimated larger reductions in coral photosynthesis (P_max_; Fig. 6e–h). The robustness shown by the winter phenotypes is even more striking considering that we applied in March relatively higher heat-stress levels, as experimental organisms were not fully acclimated in the field to control conditions (Fig. 1a). Accordingly, our study supports the relevance of the high-light photoacclimatory response of *Symbiodinium* to determine holobiont susceptibility to heat-stress, but also that this response not only relies on the genetic/functional variability of the symbiotic population, but on the seasonal condition of the host. More research is needed to understand the origin of this seasonal variability in the physiological condition of the holobiont, but also the potential contribution of the magnitude or rate of change in the local light field of *Symbiodinium in hospite* ^45, 46^ during exposure to heat stress.

Our study also indicates that caution must be taken while interpreting *F*_*v*_ */F*_*m*_ variability, in particular when it is not supported by other functional/physiological attributes. Special attention must be paid to variation in instantaneous irradiance and daily light exposure while investigating coral responses, in particular if *F*_*v*_ */F*_*m*_ is used as the main physiological descriptor of coral photosynthesis. In this respect, it should be stressed that *F*_*v*_ */F*_*m*_ variability provides more information than just description of potential adverse impacts on the photosynthetic process. For example, we observed two opposite trends in the variation of *F*_*v*_ */F*_*m*_ when examining control corals: a small but significant accumulation of photodamage in March, and a progressive recovery in October at the end of the first 10 experimental days (Fig. 6). Furthermore, in March, P_max_ of control corals from the two *Orbicella spp*. increased, whereas in October, control specimens and corals exposed to 30 °C, experienced significant P_max_ reductions despite they showed no increases in light-stress. We interpreted these findings, as the expression of the direct effect of irradiance on *Symbiodinium in hospite*, through the two opposite patterns of variation in sun’s declination that occurred in each transitional period. This *F*_*v*_ */F*_*m*_ variation of the symbionts documented more extensively by Warner *et al*.^29^ would reflect the development of the seasonal acclimatization response in the holobiont described in the present study. Inter-annual variation in the development of the summer phenotype due to different rate of change in the seasonal increase in light and temperature^47^ may affect the ability of a particular coral population to cope with similar heat-stress events.

In addition to this inter-annual variability in holobiont acclimatization, pre-exposure to moderate heat-stress has been recently documented to confer thermal tolerance^48^. In that study specimens not previously exposed to heat-stress or subjected to a previous severe event, experienced greater symbiont losses and coral cell mortality^48^. Moreover, corals previously acclimatized to “moderate heat-stress” showed similar patterns of gene expression relative to control-“non-stressed” organisms^48^. Our study supports in principle this conclusion, as corals exposed in October to 30 °C and previously acclimatized in the field to that elevated summer temperature, did not experience stress, while in March, the 30 °C treatment induced significant photodamage and reductions in coral pigmentation. However, summer acclimatization to elevated temperature produced holobionts more sensitive to lose photosynthetic performance after exposure to 32 °C, a temperature above the local MMM = 30 °C (Fig. 1a). Yet, our findings do question the conclusions of Ainsworth *et al*.^48^, and are a call of attention for the correct identification of stressed and bleached coral phenotypes. Only a deeper understanding of the physiological/cellular mechanisms involved in the acquisition of thermal tolerance will help elucidating the response of these symbioses to elevated temperature, and its true capacity to provide bleaching protection.

If we consider bleaching as the endpoint of a cascade of physiological events, which starts with an increased damage rate to the photosynthetic apparatus of *Symbiodinium* ^38–42^, resulting in increased production of ROS and cellular oxidative stress^49^, leading to the loss of host and symbiont cells through cell expulsion, apoptosis and/or necrosis^50^, and that terminates in a dysfunctional symbiosis and a dramatic loss of symbionts and photosynthetic activity, the loss of chlorophyll *a* per symbiont (Ci) could be considered one of the first regulatory mechanisms of the symbionts against the increasing levels of light-stress induced by heat-stress. When this first homeostatic response of *Symbiodinium* is able to cope with the enhancement of light-stress caused by elevated temperature (i.e., holobiont remains photosynthetically functional) the resulting loss of coral pigmentation cannot be considered “coral bleaching”, even if it results, as found in this study, in very pale corals. Therefore, the loss of chlorophyll *a* alone as well as the variation in *F*_*v*_ */F*_*m*_, are both insufficient to detect “coral bleaching” or even an “stressed coral phenotype”. A more confident descriptor is required to support the dysfunctional condition of the holobiont, particularly during experimental determinations of coral bleaching. PCA analysis performed on control and heat-stressed corals, was able to distinguish between stressed and bleached phenotypes (Fig. 3b). Bleached corals showed the highest a*_sym_ and a*_Chl*a*_ values together with the lowest holobiont photosynthetic production (P_max_, P_M_). Interestingly, some stressed but also control specimens presented high values for the optical descriptors (a*_sym_ and a*_Chl*a*_) with still significant holobiont photosynthesis. The bleached specimens of *P*. *strigosa* and *M*. *cavernosa* presenting full suppression of coral photosynthesis, showed also the largest symbiont losses (<4·10^5^ sym cm^−2^). Both species had the lowest symbiont density in control non-stressed specimens (Figs 2 and 5). Large losses of symbionts were also observed for the two *Orbicella spp*. after 20 days at 32 °C, but these experimental nubbins still retained sufficient number of symbionts (>4·10^5^ sym cm^−2^) and some photosynthetic activity at the end of the extended heat stress. The extended treatment applied only in October 2011 evidenced the accumulative nature of heat stress for these symbioses. Furthermore, it also supports the central role that light-stress plays in the determination of the severity of the impact of heat-stress and, thus, in the induction of coral bleaching.

The significant differences found between phenotypes in coral sensitivity to heat stress, may be a particular expression of a more general trade-off between performance and robustness^51^ of these ancient symbioses. Low-pigmented and low-symbiont-populated corals (here the summer phenotype) allow the establishment of more efficient and productive symbioses but concurrently more fragile under heat-stress. These holobionts are less efficient in maintaining coral performance under elevated temperature than the less productive high-pigmented and high-symbiont populated corals (here the winter phenotype). Yet, our study does not support the recent interpretation that excess of symbionts enhances coral susceptibility to bleaching^52, 53^. No physiological perturbation similar to that induced by light-stress is expected for organisms with high number of symbionts. On the contrary, a progressive reduction in the solar energy absorbed by *Symbiodinium* will occur at high symbiont densities, similarly to light-limited organisms (e.g., coral dwelling at greater depths or in shaded crevices). In our comparison, the highest number of symbionts per protein content was estimated for non-stressed organisms of *O*. *annularis*, while the lowest values were found for the species that experienced full suppression of holobiont photosynthesis (“*bleaching*”) after 20 days under severe heat-stress (32 °C), *P*. *strigosa* and *M*. *cavernosa*. The expected impact of excess of symbionts on holobiont physiology is more likely opposite to the enhancement of light-stress and photodamage on *Symbiodinium*. Higher pigment self-shading and, in consequence, reductions in photosynthetic rates similarly to deep-growing corals^54^ may affect corals with an excess of symbionts within their tissues. These organisms will only be able to sustain long periods of negative carbon balances by increasing their heterotrophic feeding^22^. In this respect, the ability of coral skeleton to enhance the local light environment of *Symbiodinium* ^31, 55^ may play a central role in the determination of the number of symbionts that a particular species can harbour. Declines in coral calcification will be also expected in highly pigmented organisms, because of the reduced photosynthetic support of coral calcification^2^.

In summary, understanding the impact of global warming on reef calcium carbonate production^56–58^ requires more attention to the direct effect of elevated temperature on coral photosynthesis, to the impact of heat-stress on the physiological coupling between coral photosynthesis and calcification, and to the contribution of an “inherent” component of the holobiont, which still needs to be elucidated, to the regulation of coral susceptibility to heat-stress.

## Material and Methods

### Description of environmental variation

Surface irradiance (E_s_, µmol quanta m^−2^ s^−1^) and seawater temperature (°C) of the reef lagoon was continuously monitored at the pier of the UASA by the Oceanographic and Meteorological Academic Service (SAMMO). Surface irradiance was measured every 15 minutes from the roof of the pier, using a cosine-corrected light sensor (LI-190R) connected to a Data Logger (LI-COR 1400, Lincoln, NE, USA). Temperature measurements were carried out daily at 9:00 a.m. from the pier for >20 years using a Mercury thermometer. During 2011 Hobo data loggers (Onset Computer Corporation, MA, USA) were installed to continuously monitor seawater temperature variation in the reef lagoon, which were located at sites where the experimental corals (nubbins) were placed for recovery after manipulation. Nubbins were place on tables, located at 5 m depth near the back-reef (SPA, 20°52'48.9” N, 86°50'59.34” W). Daily light exposure (H_day_, mol quanta m^−2^ day^−1^) was calculated as H_day_ = E_s_ · time. Monthly means of the diurnal variation in H_day_ and water temperature were calculated to describe annual fluctuations for 2011, and to estimate inter-annual variation of E_s_ for the period 2011–2014, and for water temperature for the period 1992–2015. Environmental light conditions at sampling depth in the reef lagoon (5 m) were calculated using the down-welling attenuation coefficient (K_d_, m^−1^) estimated for the reef lagoon by Enríquez and Pantoja-Reyes^59^, and confirmed for the period of sampling.

### Sample collection

Corals from three different colonies of *Orbicella annularis*, *O*. *faveolata*, *Montastraea cavernosa*, and *Pseudodiploria strigosa* were collected by Scuba at a depth of 5 m in the reef lagoon of Puerto Morelos (Cancún, Mexico), on February 23^rd^ and on September 8^th^ 2011. Samples were transported to the UASA-UNAM mesocosm facilities, and placed in outdoor tanks equipped with running seawater supplied from the reef lagoon. Tanks were shaded with neutral mesh screens, allowing for the reduction of light intensity to levels similar to the collection depth (~37% of surface irradiance, E_s_). Coral fragments were cut into equally sized experimental replicates of ~10 cm^2^ (n = 80 nubbins per species), which were fixed to PVC plates using non-toxic underwater epoxy (Z-Spar Splash Zone, A–788), and returned to the reef lagoon SPA (see previous description), where they were allowed for recovery. After 15 days, corals were transported back to the tank system, where they were distributed over three outdoor 152 L tanks supplied with running seawater (n = 25 specimens per species/per tank). The average flow rate in the experimental tanks was 1.45 L s^−1^, with a turnover rate of ∼105 min. Water temperature was maintained at 28 °C (±0.7 °C in March and ± 0.4 °C in October) using commercial aquaria heaters (Process Technology, USA), located in header tanks and connected to thermocouple sensors (J type, TEI Ingeniería, México). Water temperature in the tanks was continuously monitored with Hobo data loggers (Onset Computer Corporation, MA, USA).

Maximum photochemical efficiency of photosystem II (*F*_*v*_ */F*_*m*_) was monitored daily at dusk using a diving PAM (Heinz-Walz GmbH, Germany), as this time allows for detection of the maximum value of diurnal variation in *F*_*v*_ */F*_*m*_ (see Vásquez-Elizondo and Enríquez)^44^. Once *F*_*v*_ */F*_*m*_ stabilised (≈five days), five organisms of each species were selected randomly to perform the physiological characterisation of the initial condition of the holobiont at the beginning of each experiment. Physiological measurements took place on March 17^th^ and October 6^th^ 2011. For the characterization of coral phenotypes we used additional organisms collected in the same sampling and maintained under similar conditions. This allowed increasing the number of replicates to a maximum of 10 for *O*. *annularis*, *M*. *cavernosa* and *P*. *strigosa*, and 17 for *O*. *faveolata*, the species that showed the largest variability among replicates.

In March 2011, the experimental organisms of *O*. *faveolata* were nubbins of smaller size (~5 cm^−2^) collected in November 2010 from the same site of the reef lagoon, but prepared for another experimental purpose. These corals were maintained in the SPA during the winter 2010–2011, on the same tables, and, thus, under the same natural environmental conditions present in the reef lagoon during this period. We used these samples to reduce the impact of direct reef coral sampling, despite their remarkable differences. These organisms of *O*. *faveolata* showed unusual lower chlorophyll *a*, symbiont density and chlorophyll *a* per symbiont (Ci) in comparison with the natural population recently separated from a mother colony from the same depth in February 23^rd^ 2011 (see Figs 2 and 5). Such reduced pigmentation could be attributed to their small size and/or the large period that the nubbins were maintained on the reef (3 month), although other possible factors may also have contributed to it. To prevent misinterpretations, these organisms were not used in the characterization of coral phenotypes. However, the particular characteristics of these corals need to be considered in the interpretation of the experimental responses of *O*. *faveolata* to thermal-stress.

### Experimental analyses in mesocosms

At day 0 of the experiment, temperature for two tanks was increased to 30 °C (STD = ± 0.91 in March and ±0.58 in October), and at day 1 it was increased to 32 °C (STD = ± 0.34 in March and ±0.23 in October) in one of them. In the third tank, temperature was maintained at 28 °C (STD = ± 0.6 in March and ±0.27 in October). Light regimes were created by replacing a screening located above tanks (ML-control) by multiple screens, in order to generate three different light regimes in each tank at day 0, with an illumination of 40% (HL), 27% (ML-control) and 17% (LL) relative to surface irradiance. Accordingly, three thermal regimes (28 °C, 30 °C and 32 °C) and three light regimes (HL, ML, and LL) were applied to the experimental organisms. These experimental conditions were maintained throughout 10 days in March 2011, and 20 days in October 2011. Nubbins were shuffled randomly every day in each tank among the three light regimes after dusk, immediately after the daily *F*_*v*_ */F*_*m*_ measurements. This shuffling extended the variability of light conditions during the 10 experimental days, allowing larger fluctuations among days for diurnal light exposure, and induction of similar fluctuating light regimes between the two contrasting seasons analysed (March and October).

### Oxygen evolution determinations

Oxygen fluxes were measured polarographically, using Clark-type O_2_ electrodes (Hansatech Instruments Ltd, Norfolk, UK) connected to custom acrylic water-jacketed chambers (200 ml) filled with filtered seawater (0.45 µm). Coral samples were placed within chambers and NaHCO_3_ (5 mM) was added to prevent CO_2_ limitation during incubations^37^. Temperature within the water-jacketed chambers was maintained constant using external re-circulating water baths equipped with a temperature control system (Model AD07R–20, PolyScience, Niles, IL). O_2_ tension in the experimental chambers was maintained between 20–80% saturation by bubbling with N_2_ gas. Electrodes were calibrated with 100% oxygen saturated and with N_2_-saturated air (0% oxygen), bubbled into filtered seawater at the desired incubation temperature. Five coral samples per species were used for each physiological determination. Maximum net photosynthesis (net P_max_) was determined by exposing samples to a known saturation irradiance of 500 µmol photons m^−2^ s^−1^ for 15 minutes. Saturation irradiance was previously determined through photosynthesis response curves (P vs E) for each species. Oxygen evolution rates were measured again in darkness for five additional minutes to determine post-illumination respiration rates (R_PI_). Gross photosynthesis (P_max_) was estimated by adding O_2_ consumption through respiration to net photosynthesis (net P_max_).

### Calcification determinations

Coral calcification rates were determined following the alkalinity anomaly principle, which is based on the ratio of two equivalents of total alkalinity for each mole of CaCO_3_ precipitated. Alkalinity measurements were calculated using a modified spectrophotometer procedure (see a detailed description in Colombo-Pallota *et al*.^2^). Triplicate measurements were performed for each sample, giving a standard deviation (SD) of less than 5 µmolL^−1^ for each alkalinity measurement. Microtritration with 0.1 N HCl was conducted at a rate of 35 µL min^−1^ using a glass syringe (Hamilton Company, Reno, USA) fitted to a syringe pump (Kd Scientific Inc, Holliston, USA). Alkalinity was determined by adding an indicator, Bromocresol purple (BCP; Sigma-Aldrich), to a water sample of 5 mL before the microtritration. During titrations, water samples were gently bubbled with N_2_ and changes in absorbance were recorded spectrophotometrically at 432, 589, and 750 nm using a Miniature Ocean Optics USB4000 (Ocean Optics Ltd, Dunedin, USA). Water temperature was recorded at the end of the micro-titration with a digital thermometer. Certified reference materials of known total alkalinity (CRM, Marine Physical Laboratory, Scripps Institution of Oceanography, USA) were used to evaluate the accuracy and precision of titration analyses. Coral samples were incubated for one hour under a saturating light intensity of 500 µmol quanta cm^−2^ s^−1^, in open beakers filled with filtered oceanic seawater (50 µm) collected the same day from the Yucatan current. At the end of each incubation, 150–170 mL of water were collected and fixed with four drops of chloroform to inhibit any biological activity that could alter the alkalinity of the sample. On average, samples were stored between 1 and 14 days before alkalinity measurements were conducted. All metabolic rates were normalized to coral surface area, which was determined using the aluminium foil method. Coral tissues were covered with aluminium foil of known weight per unit area, and the amount of aluminium foil used in each determination was weighted and transformed into surface area.

### Determination of the scaling quotient of temperature (Q_10_)

For the determination of the scaling quotient of temperature (Q_10_) for coral photosynthesis, respiration, and calcification rates, coral samples were incubated in custom acrylic water-jacketed chambers for 1 hour at five temperatures, ranging from 26 °C to 34 °C at 2 °C intervals. Simultaneous determinations of coral photosynthesis and calcification were performed at 500 µmol quanta m^−2^ s^−1^. Respiration was measured for 15 min in darkness after the 1 hour light incubation. Due to the large number of incubations (n = 5 per coral species) and the time needed for each determination, each temperature increment was measured using one day for each species. After each measurement, corals were placed back into the control tank system (28 °C and ML). Each physiological determination was performed following protocols described above.

### Chlorophyll a, symbiont and protein density determinations

Chlorophyll *a* and symbiont extractions were performed by airbrushing coral samples with filtered seawater (0.45 µm) and subsequently homogenization of coral tissue slurries with a tissue homogeniser (T 10 basic Ultra-Turrax, IKA). Symbiont cells were concentrated by centrifuging for 5 min at 2000 rpm, after which 10 ml of supernatant were collected and stored at −20 °C for protein determinations. The remaining pellet was re-suspended in filtered seawater and used for chlorophyll- and symbiont density determinations. Symbiont samples were stored by adding 200 µl of iodine, and symbiont cells were later counted with a hemocytometer. Pigment extraction was performed using acetone/dimethyl sulfoxide (95:5, vol/vol). Samples were stored in darkness at 4 °C for 24 h and centrifuged before the spectrophotometric determinations. Chlorophyll determinations were measured spectrophotometrically using a Miniature Ocean Optics USB4000 (Ocean Optics Ltd, Fl) and a fixed optical geometry. For final chlorophyll *a* content calculations, we used the equations provided by Jeffrey & Humphrey^60^ for dinoflagellates. The protein content of each sample was also estimated spectrophotometrically using the equations of Whitaker & Granum^61^.

### Determination of the optical properties

Coral reflectance (R) was measured according to Enríquez *et al*.^31^ and Vásquez-Elizondo *et al*.^62^. Absorptance (A) estimations were calculated as *A* = 1−*R*, assuming that the amount of light transmitted (T) through the coral skeleton was negligible. Samples were placed in a black container with filtered seawater, illuminated with homogeneous diffuse light provided from a semi-sphere, coated with barium oxide (BaO), and placed above the sample and the black container. A submersible LED ring was placed inside the black container around the coral, and additional halogen lamps and violet-blue LEDs were used to enrich the illumination reflected by the semi-sphere in red-infrared and violet-blue regions, respectively. Reflected light was collected by placing a 2 mm diameter fiber-optic over the surface of the sample at an angle of 45°, and a distance of 1 cm from the coral surface. Measurements were performed between 400 and 750 nm using a Miniature Ocean Optics USB 4000 spectroradiometer (Ocean Optics Ltd, FL), connected to a portable computer. Calibrations were conducted using the reflectance of a bleached coral skeleton of the respective species.

The specific absorption coefficient normalized to chlorophyll *a* at 675 nm (a*_Chl*a*_, m^2^ Chl*a* ^−1^) was calculated according to Enríquez *et al*.^31^, using the equation:

1 $${a}^{* }=(De/\rho )\cdot ln(10)$$

where D_e_ is the estimated absorbance value, calculated as D_e_ = log(1/R), and ρ the chlorophyll *a* content per projected area in mg Chl*a* m^−2^. We also calculated two other descriptors for the light absorption efficiency of the holobiont, substituting ρ by (1) symbiont density (# sym cm^−2^) and (2) soluble host protein density (mg cm^−2^), respectively. The first descriptor, a*_sym_ (m^2^ sym^−1^) quantifies light absorption efficiency per symbiont; and the second, a*_M_ (cm^2^ mg protein^−1^) per protein as a proxy of holobiont mass. Both descriptors are two different proxies characterizing the contribution of each partner of this symbiosis to holobiont efficiency for solar energy collection. The second descriptor, a*_M_, follows previous suggestions by Falkowski *et al*.^27^ and previous analysis for benthic macrophytes^28^.

### Data analysis

All results are expressed as mean ± SE. Differences between phenotypes were analysed using a Welch Two Sample t-test. One-way ANOVA and Post-Hoc Tukey HSD tests were used to identify significant differences among species in the coral response to thermal-stress. Two-way ANOVA and Post-Hoc Tukey tests were used to determine the effect of temperature and time on the photochemical efficiency (*F*_*v*_ */F*_*m*_). Least-square linear regressions were used to describe the association between metabolic rates and temperature, and ANCOVA analysis allowed for the evaluation of differences among species. All analyses were conducted using R (Version 3.2.2) with the “car” (allows use of type III errors in ANOVA analysis) and “agricolae” (for use of HSD.test function) packages loaded.

## Electronic supplementary material

Supplementary Information
