# Supplementary material for: Seasonal variation modulates coral sensibility to heat-stress and explains annual changes in coral productivity
Source: Sci Rep. 2017 Jul 10;7:4937. doi: 10.1038/s41598-017-04927-8 (PMC5504023; doi:10.1038/s41598-017-04927-8)
Supplement: Supplementary file 1 — Supplementary Information [file 41598_2017_4927_MOESM1_ESM.pdf]

Manuscript: Seasonal variation modulates coral sensibility to thermal-stress and explains annual changes in coral productivity. Tim Scheufen, Wiebke E. Krämer, Roberto Iglesias-Prieto, and Susana Enríquez

Table S1: *Average values ± SE of all parameters for the winter and summer phenotypes. Different letters indicate differences among species within one phenotype (one-way ANOVA, P<0.05, Tukey Post-hoc P<0.05), while asterisks indicate differences between phenotypes (Student T-Test, P<0.05).*

Parameter		*O. annularis*	*O. faveolata*	*M. cavernosa*	*P. strigosa*
Chlorophyll *a* density	Winter	82.32 ± 12.16^{a}	$189.1 \pm 14.91^{b*}$	116.81 ± 10.09^{a}	$121.8 \pm 18.3^{a*}$
(mg Chl*a* m^{-2})	Summer	$79.69 \pm 11.6^{a/c}$	$144.96 \pm 4.44^{b*}$	100.59 ± 3.75^{a}	$59.47 \pm 3.6^{c*}$
Symbiont density	Winter	$3.26 \pm 0.4^{*}$	3.14 ± 0.26	2.01 ± 0.11	2.26 ± 0.47
($x10^6$ # sym cm^{-2})	Summer	$2.03 \pm 0.19^{a*}$	3.36 ± 0.12^{b}	$1.79 \pm 0.2^{a/c}$	1.26 ± 0.09^{c}
Ci	Winter	$2.57 \pm 0.19^{a*}$	$6.61 \pm 0.69^{b*}$	5.83 ± 0.44^{b}	6.2 ± 0.75^{b}
(pg Chl*a* sym^{-1})	Summer	$3.88 \pm 0.26^{a*}$	$4.32 \pm 0.05^{a/b*}$	5.89 ± 0.6^{b}	$4.8 \pm 0.36^{a/b}$
Host soluble protein	Winter	3.85 ± 0.83^{a}	7.9 ± 0.8^{b}	$13.1 \pm 0.66^{c*}$	$7.06 \pm 0.92^{a/b}$
(mg protein cm^{-2})	Summer	4.1 ± 0.32^{a}	6.3 ± 0.58^{b}	$10.81 \pm 0.42^{c*}$	$5.68 \pm 0.6^{a/b}$
Symbiont content per host protein	Winter	$0.83 \pm 0.07^{a*}$	0.36 ± 0.04^{b}	0.15 ± 0.01^{c}	$0.26 \pm 0.06^{b/c}$
(# sym ng $protein^{-1}$)	Summer	$0.49 \pm 0.01^{a*}$	0.55 ± 0.05^{a}	0.17 ± 0.02^{b}	0.23 ± 0.02^{b}
Calcification, G_{max}	Winter	$0.26 \pm 0.06^{a/b}$	0.33 ± 0.04^{a}	$0.09 \pm 0.03^{b*}$	0.36 ± 0.05^{a}
(µmol $CaCO_3$ cm^{-2} h^{-1})	Summer	0.41 ± 0.17^{a}	0.69 ± 0.28^{a}	$0.38 \pm 0.03^{a*}$	0.36 ± 0.07^{a}
P_{max}	Winter	$2.78 \pm 0.14^{*}$	$2.58 \pm 0.22^{*}$	$2.54 \pm 0.27^{*}$	$2.7 \pm 0.14^{*}$
(µmol O_2 cm^{-2} h^{-1})	Summer	$5.25 \pm 0.58^{a*}$	$5.71 \pm 0.53^{a*}$	$3.48 \pm 0.09^{b*}$	$4.5 \pm 0.43^{a/b*}$
Respiration	Winter	1.17 ± 0.1^{a}	0.88 ± 0.1^{a}	1.05 ± 0.15^{a}	0.9 ± 0.15^{a}
(µmol O_2 cm^{-2} h^{-1})	Summer	0.93 ± 0.1^{a}	1.47 ± 0.21^{a}	1.03 ± 0.09^{a}	1.63 ± 0.29^{a}
P_{sym}	Winter	$1.04 \pm 0.18^{a/b*}$	$0.86 \pm 0.08^{a*}$	$1.31 \pm 0.13^{a/b*}$	$1.63 \pm 0.3^{b*}$
(pmol O_2 sym^{-1} h^{-1})	Summer	$2.57 \pm 0.07^{a/b*}$	$1.72 \pm 0.21^{a*}$	$2.04 \pm 0.22^{a*}$	$3.69 \pm 0.48^{b*}$
P_M	Winter	0.81 ± 0.2^{a}	$0.33 \pm 0.03^{b*}$	$0.17 \pm 0.02^{b*}$	$0.39 \pm 0.07^{b*}$
(µmol O_2 $protein^{-1}$ h^{-1})	Summer	1.27 ± 0.07^{a}	$0.95 \pm 0.17^{a/b*}$	$0.32 \pm 0.2^{c*}$	$0.83 \pm 0.12^{b*}$
Absorptance	Winter	0.93 ± 0.008^{a}	$0.92 \pm 0.005^{a*}$	$0.91 \pm 0.008^{a/b*}$	0.89 ± 0.012^{b}
	Summer	$0.93 \pm 0.008^{a/c}$	$0.84 \pm 0.019^{b*}$	$0.94 \pm 0.004^{a*}$	0.89 ± 0.01^{c}
a^*_{Chla}	Winter	0.033 ± 0.003^{a}	0.015 ± 0.002^{b}	0.023 ± 0.003^{b}	$0.021 \pm 0.003^{b*}$
(m^2 mg Chla^{-1})	Summer	0.036 ± 0.004^{a}	0.013 ± 0.001^{b}	0.028 ± 0.001^{a}	$0.035 \pm 0.005^{a*}$
a^*_{sym}	Winter	0.008 ± 0.001	$0.0076 \pm 0.001^{*}$	0.01 ± 0.0009	0.0108 ± 0.0016
(m^2 sym^{-1})	Summer	$0.01 \pm 0.0006^{a/b}$	$0.0046 \pm 0.0002^{b*}$	0.0139 ± 0.0018^{a}	0.0139 ± 0.0013^{a}
a^*_M	Winter	0.07 ± 0.014^{a}	0.03 ± 0.003^{b}	$0.02 \pm 0.001^{b*}$	0.03 ± 0.004^{b}
(m^2 mg $protein^{-1}$)	Summer	0.05 ± 0.003^{a}	0.02 ± 0.003^{b}	$0.02 \pm 0.001^{b*}$	0.03 ± 0.005^{b}

Manuscript: Seasonal variation modulates coral sensibility to thermal-stress and explains annual changes in coral productivity. Tim Scheufen, Wiebke E. Krämer, Roberto Iglesias-Prieto, and Susana Enríquez

Table S2: Student t-test analysis comparing the structural and the functional descriptors of the winter and summer phenotypes of the four coral species investigated. Significant values (p<0.05) are marked in bold.

Species	Parameter	df	t	p	Parameter	df	t	p
O. annularis	Chlorophyll *a* density	9	0.156	0.879	Respiration	9	1.684	0.124
O. faveolata	(mg Chl*a* m^{-2})	18	2.838	**<0.05**	(µmol O_2 cm^{-2} h^{-1})	4	-2.571	0.058
M. cavernosa		11	1.506	0.16		12	0.121	0.905
P. strigosa		9	3.342	**<0.01**		6	-2.19	0.069
O. annularis	Symbiont density	11	2.778	**<0.05**	P_{sym}	11	-7.965	**<0.001**
O. faveolata	($x10^6$ # sym cm^{-2})	18	-0.746	0.465	(pmol O_2 sym^{-1} h^{-1})	4	-3.892	**<0.05**
M. cavernosa		6	0.959	0.371		6	-2.919	**<0.05**
P. strigosa		9	2.1	0.063		7	-3.673	**<0.01**
O. annularis	Ci	6	-4.014	**<0.01**	P_M	4	-2.173	0.083
O. faveolata	(pg Chl*a* sym^{-1})	16	3.326	**<0.01**	(µmol O_2 mg^{-1} protein h^{-1})	3	-3.528	**<0.05**
M. cavernosa		8	-0.085	0.935		7	-6.507	**<0.001**
P. strigosa		12	1.683	0.118		6	-3.14	**<0.05**
O. annularis	Host soluble protein	5	-0.28	0.791	Absorptance	9	-0.365	0.724
O. faveolata	(mg protein cm^{-2})	9	1.626	0.135		3	4.268	**<0.05**
M. cavernosa		6	2.927	**<0.05**		12	-3.332	**<0.01**
P. strigosa		6	1.26	0.249		12	-0.032	0.975
O. annularis	Symbiont content per	4	4.898	**<0.01**	a^*_{Chla}	7	-0.411	0.693
O. faveolata	host protein	6	-2.973	**<0.05**	(m^2 mg $Chla^{-1}$)	18	1.51	0.148
M. cavernosa	(# sym ng $protein^{-1}$)	5	-0.524	0.619		11	-1.879	0.086
P. strigosa		5	0.472	0.656		6	-2.582	**<0.05**
O. annularis	Calcification, G_{max}	3	-0.826	0.458	a^*_{sym}	11	-1.591	0.139
O. faveolata	(µmol $CaCO_3$ cm^{-2} h^{-1})	3	-1.237	0.3	(m^2 sym^{-1})	18	6.127	**<0.001**
M. cavernosa		7	-6.232	**<0.001**		6	-1.858	0.11
P. strigosa		6	-0.034	0.974		12	-1.449	0.172
O. annularis	P_{max}	3	-4.149	**<0.05**	a^*_M	4	1.265	0.27
O. faveolata	(µmol O_2 cm^{-2} h^{-1})	4	-5.468	**<0.01**	(m^2 mg $protein^{-1}$)	9	0.583	0.573
M. cavernosa		9	-3.286	**<0.01**		7	-4.212	**<0.05**
P. strigosa		4	-3.998	**<0.05**		7	0.033	0.975

Manuscript: Seasonal variation modulates coral sensibility to thermal-stress and explains annual changes in coral productivity. Tim Scheufen, Wiebke E. Krämer, Roberto Iglesias-Prieto, and Susana Enríquez

Table S3: One-way ANOVA analysis comparing the structural and the functional descriptors of the four species within the two phenotypes. Significant values (p<0.05) are marked in bold.

Parameter	Phenotype	df	SM	F	P
Chlorophyll *a* density	Winter	3,43	82423	10.447	**<0.001**
(mg Chl*a* m^{-2})	Summer	3,14	17380.8	33.801	**<0.001**
Symbiont density	Winter	3,43	13.092	3.4963	**<0.05**
($x10^6$ # sym cm^{-2})	Summer	3,14	10.2967	30.477	**<0.001**
Ci	Winter	3,43	112.606	8.0763	**<0.001**
(pg Chl*a* sym^{-1})	Summer	3,14	10.254	4.3749	**<0.05**
Host soluble protein	Winter	3,19	220.597	18.625	**<0.001**
(mg protein cm^{-2})	Summer	3,14	116.56	34.282	**<0.001**
Symbiont content per host protein (# sym ng $protein^{-1}$)	Winter	3,19	1.3501	34.987	**<0.001**
	Summer	3,14	0.47173	39.371	**<0.001**
Calcification, G_{max}	Winter	3,15	0.152	4.761	**<0.05**
(µmol $CaCO_3$ cm^{-2} h^{-1})	Summer	3,14	0.30575	0.983	0.43
P_{max}	Winter	3,41	0.369	0.2468	0.86
(µmol O_2 cm^{-2} h^{-1})	Summer	3,14	12.813	5.3382	**<0.05**
Respiration	Winter	3,42	0.6281	1.1883	0.33
(µmol O_2 cm^{-2} h^{-1})	Summer	3,14	1.5656	2.8783	0.07
P_{sym}	Winter	3,41	4.0026	3.9562	**<0.05**
(pmol O_2 sym^{-1} h^{-1})	Summer	3,14	10.5553	8.1512	**<0.01**
P_M	Winter	3,18	1.1533	7.23	**<0.01**
(µmol O_2 $protein^{-1}$ h^{-1})	Summer	3,14	2.1227	13.723	**<0.001**
Absorptance	Winter	3,43	0.0093	4.2229	**<0.05**
	Summer	3,14	0.0283	17.279	**<0.001**
a^*_{Chla}	Winter	3,43	0.00404	7.6746	**<0.001**
(m^2 mg $Chla^{-1}$)	Summer	3,14	0.0015	9.4304	**<0.01**
a^*_{sym}	Winter	3,43	0.000097	2.6417	0.06
(m^2 sym^{-1})	Summer	3,14	0.000255	11.675	**<0.001**
a^*_M	Winter	3,19	0.0076	9.1048	**<0.001**
(m^2 mg $protein^{-1}$)	Summer	3,14	0.001885	11.842	**<0.001**

Manuscript: Seasonal variation modulates coral sensibility to thermal-stress and explains annual changes in coral productivity. Tim Scheufen, Wiebke E. Krämer, Roberto Iglesias-Prieto, and Susana Enríquez

Table S4: Least-square regression analyses for the description of the temperature scaling factor, Q10, of each metabolic rate for the four coral species analyzed. Differences between slopes of each season are indicated with different letters according to the results shown in Table S4.

Metabolic rate	Species		Slope	RSD	R^2	P	n
P_{max} (μmol O_2 cm^{-2} h^{-1})	*O. annularis*	Winter	0.132 ± 0.038^a	0.168	0.792	0.07	5
		Summer	0.262 ± 0.006^b	0.026	0.999	**<0.001**	5
	O. faveolata	Winter	0.155 ± 0.024^a	0.106	0.933	**<0.05**	5
		Summer	0.263 ± 0.008^b	0.036	0.997	**<0.001**	5
	M. cavernosa	Winter	0.125 ± 0.017^a	0.078	0.943	**<0.05**	5
		Summer	0.162 ± 0.001^b	0.005	0.999	**<0.001**	5
	P. strigosa	Winter	0.039 ± 0.005^a	0.021	0.958	**<0.05**	5
		Summer	0.188 ± 0.002^b	0.008	0.999	**<0.001**	5
Respiration (μmol O_2 cm^{-2} h^{-1})	*O. annularis*	Winter	0.066 ± 0.006^a	0.027	0.976	**<0.01**	5
		Summer	0.159 ± 0.015^b	0.066	0.975	**<0.01**	5
	O. faveolata	Winter	0.07 ± 0.003^a	0.014	0.994	**<0.01**	5
		Summer	0.159 ± 0.008^b	0.038	0.992	**<0.01**	5
	M. cavernosa	Winter	0.049 ± 0.006^a	0.025	0.961	**<0.05**	5
		Summer	0.1 ± 0.001^b	0.006	0.999	**<0.001**	5
	P. strigosa	Winter	0.022 ± 0.005^a	0.022	0.859	**<0.05**	5
		Summer	0.113 ± 0.004^b	0.019	0.996	**<0.01**	5
Photosynthesis-Respiration ratio (P/R)	*O. annularis*	Winter	-0.086 ± 0.008^a	0.037	0.973	**<0.01**	5
		Summer	-0.166 ± 0.007^b	0.029	0.995	**<0.001**	5
	O. faveolata	Winter	-0.053 ± 0.016^a	0.071	0.774	0.078	5
		Summer	-0.144 ± 0.014^b	0.064	0.971	**<0.01**	5
	M. cavernosa	Winter	-0.052 ± 0.009^a	0.038	0.924	**<0.05**	5
		Summer	-0.09 ± 0.009^b	0.039	0.972	**<0.01**	5
	P. strigosa	Winter	-0.074 ± 0.007^a	0.031	0.974	**<0.01**	5
		Summer	-0.116 ± 0.004^b	0.02	0.996	**<0.01**	5
Calcification (G_{max}) (μmol $CaCO_3$ cm^{-2} h^{-1})	*O. annularis*	Winter	0.024 ± 0.003^a	0.013	0.957	**<0.05**	5
		Summer	0.048 ± 0.021^b	0.095	0.57	0.155	5
	O. faveolata	Winter	0.029 ± 0.007	0.03	0.857	**<0.05**	5
		Summer	-0.007 ± 0.05	0.242	0	0.907	5
	M. cavernosa	Winter	0.021 ± 0.003	0.013	0.942	**<0.05**	5
		Summer	0.03 ± 0.015	0.068	0.492	0.187	5
	P. strigosa	Winter	0.007 ± 0.007	0.03	0.065	0.386	5
		Summer	-0.2 ± 0.043	0.194	0	0.683	5

Manuscript: Seasonal variation modulates coral sensibility to thermal-stress and explains annual changes in coral productivity. Tim Scheufen, Wiebke E. Krämer, Roberto Iglesias-Prieto, and Susana Enríquez

Table S5*: ANCOVA analysis comparing differences among phenotypes in the slopes estimated for the temperature scaling factor, Q10, of each metabolic rate of the four coral species analysed.*

Metabolic rate	Species	df	MS	F	P
P_{max} ($\mu mol\ O_2\ cm^{-2}\ h^{-1}$)	*O. annularis*	3,4	0.0072	73.776	**<0.001**
	O. faveolata	3,4	0.0031	157.96	**<0.001**
	M. cavernosa	3,4	0.0015	128.529	**<0.001**
	P. strigosa	3,4	0.0001	6001.677	**<0.001**
Respiration ($\mu mol\ O_2\ cm^{-2}\ h^{-1}$)	*O. annularis*	3,4	0.0013	97.255	**<0.001**
	O. faveolata	3,4	0.0004	288.239	**<0.001**
	M. cavernosa	3,4	0.0002	393.244	**<0.001**
	P. strigosa	3,4	0.0002	584.319	**<0.001**
Photosynthesis-Respiration ratio (P/R)	*O. annularis*	3,4	0.0006	226.363	**<0.001**
	O. faveolata	3,4	0.0023	38.818	**<0.001**
	M. cavernosa	3,4	0.0008	89.683	**<0.001**
	P. strigosa	3,4	0.0003	205.831	**<0.001**
Calcification (G_{max}) ($\mu mol\ CaCO_3\ cm^{-2}\ h^{-1}$)	*O. annularis*	3,4	0.0023	10.281	**<0.05**
	O. faveolata	3,4	0.0149	1.098	0.447
	M. cavernosa	3,4	0.0012	4.573	0.088
	P. strigosa	3,4	0.0096	0.592	0.652

Manuscript: Seasonal variation modulates coral sensibility to thermal-stress and explains annual changes in coral productivity. Tim Scheufen, Wiebke E. Krämer, Roberto Iglesias-Prieto, and Susana Enríquez

Table S6: *One-way ANOVA test for the analysis of the effect of the three temperature treatments applied in March 2011 on the different descriptors of the winter phenotype of the four coral species investigated*

		df	SM	F	P
Chlorophyll a density	*O. annularis*	3,16	10981.85	4.823	**<0.05**
(mg Chl*a* m^{-2})	*O. faveolata*	3,16	7125.535	8.611	**<0.05**
	M. cavernosa	3,16	15235.19	3.3	**<0.05**
	P. strigosa	3,16	11705.79	9.349	**<0.05**
Symbiont density	*O. annularis*	3,16	11.137	3.578	**<0.05**
(x10^{6} # sym cm^{-2})	*O. faveolata*	3,16	3.237	3.736	**<0.05**
	M. cavernosa	3,16	1.004	1.254	0.323
	P. strigosa	3,16	1.103	4.971	**<0.05**
Ci	*O. annularis*	3,16	1.518	1.705	0.206
(pg Chl*a* sym^{-1})	*O. faveolata*	3,16	5.934	3.373	**<0.05**
	M. cavernosa	3,14	16.934	2.572	0.096
	P. strigosa	3,16	15.229	1.974	0.159
Host soluble protein	*O. annularis*	3,15	9.307	1.005	0.418
(mg protein cm^{-2})	*O. faveolata*	3,16	6.969	0.47	0.707
	M. cavernosa	3,16	167.748	1.518	0.248
	P. strigosa	3,16	39.123	1.335	0.298
Symbiont content per host protein	*O. annularis*	3,15	0.76	4.844	**<0.05**
(# sym ng protein^{-1})	*O. faveolata*	3,16	0.18	3.632	**<0.05**
	M. cavernosa	3,16	0.026	1.654	0.217
	P. strigosa	3,16	0.133	1.461	0.263
P_{max}	*O. annularis*	3,14	3.684	7.371	**<0.05**
(µmol O_2 cm^{-2} h^{-1})	*O. faveolata*	3,14	1.923	2.188	0.135
	M. cavernosa	3,13	1.116	2.1	0.15
	P. strigosa	3,15	4.875	9.86	**<0.05**
P_{sym}	*O. annularis*	3,14	0.428	0.559	0.651
(pmol O_2 sym^{-1} h^{-1})	*O. faveolata*	3,14	2.085	1.683	0.216
	M. cavernosa	3,13	0.135	0.54	0.663
	P. strigosa	3,15	1.076	1.826	0.186
P_M	*O. annularis*	3,13	0.625	1.866	0.185
(µmol O_2 protein^{-1} h^{-1})	*O. faveolata*	3,14	0.045	0.414	0.746
	M. cavernosa	3,13	0.015	0.8	0.516
	P. strigosa	3,15	0.153	1.601	0.231

Manuscript: Seasonal variation modulates coral sensibility to thermal-stress and explains annual changes in coral productivity. Tim Scheufen, Wiebke E. Krämer, Roberto Iglesias-Prieto, and Susana Enríquez

Table S7: *Average values ± SE of all parameters for the response to thermal-stress of the winter phenotypes. Different letters indicate significant differences between temperature treatments within each species (one-way ANOVA, P<0.05, Tukey Post-hoc P<0.05).*

		Day 0	Day 10		
		28ºC	28ºC	30ºC	32ºC
Chlorophyll *a* density (mg Chl*a* m^{-2})	*O. annularis*	94 ± 22[a]	85 ± 5[a]	69 ± 9[a/b]	33 ± 3[b]
	O. faveolata	73 ± 9[a]	80 ± 5[a]	40 ± 8[b]	38 ± 8[b]
	M. cavernosa	139 ± 8[a/b]	181 ± 24[a]	142 ± 14[a/b]	103 ± 2[b]
	P. strigosa	86 ± 4[a/b]	112 ± 15[a]	65 ± 5[b/c]	47 ± 8[c]
Symbiont density ($x10^6$ # sym cm^{-2})	*O. annularis*	3.31 ± 0.79[a]	2.65 ± 0.29[a/b]	2.23 ± 0.33[a/b]	1.25 ± 0.12[b]
	O. faveolata	1.9 ± 0.29[a/b]	2.46 ± 0.17[a]	1.6 ± 0.21[a/b]	1.39 ± 0.27[b]
	M. cavernosa	2 ± 0.1	1.82 ± 0.4	1.59 ± 0.16	1.41 ± 0.13
	P. strigosa	1.64 ± 0.19[a]	1.74 ± 0.08[a]	1.38 ± 0.07[a/b]	1.13 ± 0.1[b]
Ci (pg Chl*a* sym^{-1})	*O. annularis*	2.94 ± 0.21	3.39 ± 0.41	3.14 ± 0.09	2.63 ± 0.14
	O. faveolata	3.94 ± 0.33[a]	3.31 ± 0.27[b/c]	2.49 ± 0.4[c]	2.83 ± 0.37[b/c]
	M. cavernosa	7 ± 0.38	9.49 ± 0.63	7.73 ± 0.88	7.11 ± 0.83
	P. strigosa	5.66 ± 1	6.38 ± 0.76	4.69 ± 0.26	4.11 ± 0.64
Host soluble protein (mg protein cm^{-2})	*O. annularis*	3.85 ± 0.83	4.03 ± 0.72	5.6 ± 0.49	4.66 ± 1.2
	O. faveolata	7.9 ± 0.8	6.06 ± 1.08	5.89 ± 0.66	7.41 ± 1.16
	M. cavernosa	14.84 ± 2.23	13.86 ± 3.94	15.1 ± 2.38	8 ± 1.81
	P. strigosa	7.06 ± 0.92	7.53 ± 1.5	4.21 ± 1.14	7.63 ± 1.85
Symbiont content per host protein (# sym ng $protein^{-1}$)	*O. annularis*	0.83 ± 0.07[a]	0.74 ± 0.13[a/b]	0.4 ± 0.05[b]	0.38 ± 0.16[b]
	O. faveolata	0.33 ± 0.06[a/b]	0.46 ± 0.09[a]	0.28 ± 0.04[a/b]	0.2 ± 0.03[b]
	M. cavernosa	0.14 ± 0.02	0.17 ± 0.04	0.11 ± 0.02	0.21 ± 0.04
	P. strigosa	0.26 ± 0.06	0.28 ± 0.06	0.43 ± 0.1	0.2 ± 0.08

Manuscript: Seasonal variation modulates coral sensibility to thermal-stress and explains annual changes in coral productivity. Tim Scheufen, Wiebke E. Krämer, Roberto Iglesias-Prieto, and Susana Enríquez

P_{max} (μmol O_2 cm^{-2} h^{-1})	*O. annularis*	2.52 ± 0.16[a]	3.34 ± 0.21[b]	2.51 ± 0.16[a]	2.03 ± 0.28[a]
	O. faveolata	2.37 ± 0.3	3.4 ± 0.18	2.99 ± 0.25	2.44 ± 0.14
	M. cavernosa	2.16 ± 0.23	1.62 ± 0.19	1.57 ± 0.18	1.65 ± 0.14
	P. strigosa	2.55 ± 0.12[a]	2.12 ± 0.14[a/b]	1.65 ± 0.14[b/c]	1.15 ± 0.34[c]
P_{sym} (pmol O_2 sym^{-1} h^{-1})	*O. annularis*	1.06 ± 0.33	1.31 ± 0.14	1.22 ± 0.17	1.53 ± 0.25
	O. faveolata	0.91 ± 0.11	1.43 ± 0.17	1.97 ± 0.27	2.33 ± 0.77
	M. cavernosa	1.08 ± 0.11	1.19 ± 0.22	1 ± 0.1	1.24 ± 0.11
	P. strigosa	1.65 ± 0.23	1.23 ± 0.11	1.22 ± 0.14	0.98 ± 0.32
P_M (μmol O_2 $protein^{-1}$ h^{-1})	*O. annularis*	0.81 ± 0.2	0.94 ± 0.17	0.46 ± 0.03	0.78 ± 0.11
	O. faveolata	0.5 ± 0.1	0.6 ± 0.06	0.52 ± 0.06	0.47 ± 0.17
	M. cavernosa	0.15 ± 0.02	0.17 ± 0.06	0.11 ± 0.02	0.2 ± 0.05
	P. strigosa	0.39 ± 0.07	0.34 ± 0.08	0.47 ± 0.07	0.21 ± 0.12

Manuscript: Seasonal variation modulates coral sensibility to thermal-stress and explains annual changes in coral productivity. Tim Scheufen, Wiebke E. Krämer, Roberto Iglesias-Prieto, and Susana Enríquez

Table S8: *One-way ANOVA test for the analysis of the effect of the three temperature treatments applied in October 2011 on the different descriptors of the summer phenotype of the four coral species investigated*

		df	SM	F	P
Chlorophyll *a* density	*O. annularis*	6,31	45745.05	12	**<0.05**
(mg Chl*a* m^{-2})	*O. faveolata*	6,34	93885.73	30.471	**<0.05**
	M. cavernosa	6,36	124364.9	90.22	**<0.05**
	P. strigosa	6,23	32450.04	29.26	**<0.05**
Symbiont density	*O. annularis*	6,29	21.162	19.36	**<0.05**
($x10^6$ # sym cm^{-2})	*O. faveolata*	6,34	54.133	48.375	**<0.05**
	M. cavernosa	6,36	27.641	51.238	**<0.05**
	P. strigosa	6,24	19.756	26.846	**<0.05**
Ci	*O. annularis*	6,29	28.888	2.075	0.087
(pg Chl*a* sym^{-1})	*O. faveolata*	6,34	24.905	3.688	**<0.05**
	M. cavernosa	6,36	10.755	0.626	0.709
	P. strigosa	6,23	22.696	1.629	0.184
Host soluble protein	*O. annularis*	6,31	58.933	8.333	**<0.05**
(mg protein cm^{-2})	*O. faveolata*	6,34	31.351	1.976	0.097
	M. cavernosa	6,36	200.202	19.172	**<0.05**
	P. strigosa	6,24	17.005	1.774	0.147
Symbiont content per host protein	*O. annularis*	6,29	1.242	20.215	**<0.05**
(# sym ng $Protein^{-1}$)	*O. faveolata*	6,34	2.04	26.34	**<0.05**
	M. cavernosa	6,36	0.364	31.244	**<0.05**
	P. strigosa	6,24	0.997	10.156	**<0.05**
P_{max}	*O. annularis*	5,15	45.332	10.102	**<0.05**
(µmol O_2 cm^{-2} h^{-1})	*O. faveolata*	5,15	56.77	24.954	**<0.05**
	M. cavernosa	5,16	33.296	66.304	**<0.05**
	P. strigosa	5,16	41.276	28.138	**<0.05**
P_{sym}	*O. annularis*	5,14	2.126	1.054	0.426
(pmol O_2 sym^{-1} h^{-1})	*O. faveolata*	5,15	3.246	3.458	**<0.05**
	M. cavernosa	5,16	6.572	13.41	**<0.05**
	P. strigosa	5,16	19.866	6.682	**<0.05**
P_M	*O. annularis*	5,15	3.695	14.242	**<0.05**
(µmol O_2 $protein^{-1}$ h^{-1})	*O. faveolata*	5,15	2.264	7.736	**<0.05**
	M. cavernosa	5,16	0.425	20.424	**<0.05**
	P. strigosa	5,16	1.57	7.927	**<0.05**

Manuscript: Seasonal variation modulates coral sensibility to thermal-stress and explains annual changes in coral productivity. Tim Scheufen, Wiebke E. Krämer, Roberto Iglesias-Prieto, and Susana Enríquez

Table S9: *Average values ± SE of all parameters for the response to thermal-stress of the summer phenotypes. Different letters indicate differences between temperature treatments within each species (one-way ANOVA, P<0.05, Tukey Post-hoc P<0.05).*

		Day 0	Day 10			Day 20		
		28ºC	28ºC	30ºC	32ºC	28ºC	30ºC	32ºC
Chlorophyll *a* density (mg Chl*a* m^{-2})	*O. annularis*	80 ± 12^{a}	99 ± 7^{a}	105 ± 26^{a}	66 ± 30$^{a/b}$	110.01 ± 11.6^{a}	107.15 ± 3.17^{a}	29.78 ± 5.01^{b}
	O. faveolata	145 ± 4$^{a/b}$	151 ± 13^{a}	141 ± 11$^{a/b}$	92 ± 26$^{b/c}$	169.3 ± 3.31^{a}	126.92 ± 4.14$^{a/b}$	49.99 ± 5.98^{c}
	M. cavernosa	101 ± 4$^{a/c}$	131 ± 25$^{a/b}$	136 ± 17^{b}	71 ± 3^{c}	129.3 ± 4.9$^{a/b}$	123.33 ± 8.6$^{a/b}$	12.13 ± 1.29^{d}
	P. strigosa	59 ± 4^{a}	72 ± 5$^{a/b}$	101 ± 3^{b}	51 ± 10^{a}	103.7 ± 10.36^{b}	88.13 ± 6.55^{b}	10.9 ± 2.27^{c}
Symbiont density (x10^{6} # sym cm^{-2})	*O. annularis*	2.03 ± 0.19$^{a/b}$	2.10 ± 0.13$^{a/b}$	2.31 ± 0.35^{a}	1.13 ± 0.52$^{b/c}$	2.3 ± 0.2^{a}	2.06 ± 0.21$^{a/b}$	0.55 ± 0.07^{c}
	O. faveolata	3.36 ± 0.12^{a}	3.3 ± 0.46^{a}	2.71 ± 0.41^{a}	1.59 ± 0.32^{b}	3.48 ± 0.16^{a}	2.68 ± 0.15^{a}	0.79 ± 0.09^{b}
	M. cavernosa	1.79 ± 0.2^{a}	2.02 ± 0.25^{a}	1.87 ± 0.22^{a}	1.41 ± 0.2^{a}	1.92 ± 0.14^{a}	1.78 ± 0.21^{a}	0.21 ± 0.03^{b}
	P. strigosa	1.26 ± 0.09^{a}	1.51 ± 0.19$^{a/b}$	2.1 ± 0.48$^{b/c}$	1.08 ± 0.11^{a}	2.12 ± 0.19$^{b/c}$	2.41 ± 0.12^{c}	0.19 ± 0.03^{d}
Ci (pg Chl*a* sym^{-1})	*O. annularis*	3.88 ± 0.26	4.68 ± 0.08	4.39 ± 0.5	5.76 ± 0.15	4.79 ± 0.29	5.42 ± 0.53	6.39 ± 0.61
	O. faveolata	4.32 ± 0.05^{a}	4.66 ± 0.36$^{a/b}$	5.33 ± 0.51$^{a/b}$	5.55 ± 0.63$^{a/b}$	4.9 ± 0.23$^{a/b}$	4.76 ± 0.19$^{a/b}$	6.34 ± 0.32^{b}
	M. cavernosa	5.89 ± 0.61	6.5 ± 0.79	7.37 ± 0.96	5.27 ± 0.74	6.8 ± 0.24	7.06 ± 0.36	6.48 ± 0.48
	P. strigosa	4.8 ± 0.36	4.98 ± 0.94	5.41 ± 1.36	4.62 ± 0.47	4.93 ± 0.31	3.67 ± 0.23	6.45 ± 1
Host soluble protein (mg protein cm^{-2})	*O. annularis*	4.1 ± 0.32$^{a/b}$	3.24 ± 0.12^{b}	4.5 ± 0.97$^{a/b}$	7.7 ± 1.32^{c}	4.36 ± 0.37$^{a/b}$	5.52 ± 0.58$^{a/c}$	3.36 ± 0.22^{b}
	O. faveolata	6.3 ± 0.58	7.2 ± 1.4	4.95 ± 0.98	8.72 ± 1.98	5.27 ± 0.81	6.1 ± 0.59	6.09 ± 0.28
	M. cavernosa	10.81 ± 0.42^{a}	7.84 ± 0.87$^{a/b/c}$	8.79 ± 0.93$^{a/b/c}$	14.11 ± 0.21^{d}	9.22 ± 0.18$^{a/b}$	7.56 ± 0.8$^{b/c}$	6.53 ± 0.32^{c}
	P. strigosa	5.68 ± 0.6	6.17 ± 0.79	5.25 ± 0.38	6.91 ± 0.2	5.12 ± 0.73	4.86 ± 0.64	4.45 ± 0.43
Symbiont content per host protein (# sym ng $protein^{-1}$)	*O. annularis*	0.49 ± 0.01$^{a/b}$	0.65 ± 0.06^{a}	0.54 ± 0.06$^{a/b}$	0.13 ± 0.05^{c}	0.54 ± 0.06$^{a/b}$	0.39 ± 0.06^{b}	0.17 ± 0.03^{c}
	O. faveolata	0.55 ± 0.05$^{a/b}$	0.47 ± 0.05$^{a/b/c}$	0.56 ± 0.03$^{a/b}$	0.21 ± 0.08$^{c/d}$	0.73 ± 0.12^{a}	0.45 ± 0.04$^{b/c}$	0.13 ± 0.01^{d}
	M. cavernosa	0.17 ± 0.02$^{a/b}$	0.27 ± 0.06^{a}	0.22 ± 0.04^{a}	0.1 ± 0.02$^{b/c}$	0.21 ± 0.02^{a}	0.24 ± 0.03^{a}	0.03 ± 0^{c}

	P. strigosa	0.23±0.02$^{a/b/c}$	0.26 ± 0.05$^{a/b/c/d}$	0.41 ± 0.1^{d}	0.16 ± 0.01$^{b/c}$	0.46 ± 0.08$^{a/d}$	0.54 ± 0.09^{d}	0.05 ± 0.01^{c}
P_{max} (μmol O_2 cm^{-2} h^{-1})	*O. annularis*	5.25 ± 0.58^{a}	3.91 ± 0.39$^{a/b}$	4.21 ± 0.18$^{a/b}$	2.37 ± 1.13$^{b/c}$	3.91 ± 0.39$^{a/b}$		0.54 ± 0.33^{c}
	O. faveolata	5.71 ± 0.53^{a}	3.24 ± 0.14^{b}	5.15 ± 0.18^{a}	2.13 ± 0.72$^{b/c}$	3.24 ± 0.14^{b}		0.73 ± 0.09^{c}
	M. cavernosa	3.48 ± 0.09^{a}	3.11 ± 0.47$^{a/b}$	2.73 ± 0.12^{b}	0.85 ± 0.09^{c}	3.24 ± 0.14$^{a/b}$		0.1 ± 0.03^{c}
	P. strigosa	4.5 ± 0.43^{a}	3.14 ± 0.27^{b}	3.02 ± 0^{b}	1.42 ± 0.31^{c}	3.14 ± 0.27^{b}		0.22 ± 0.08^{c}
P_{sym} (pmol O_2 sym^{-1} h^{-1})	*O. annularis*	2.57 ± 0.07	1.86 ± 0.15	1.9 ± 0.26	1.73 ± 0.63	1.75 ± 0.16		1.64 ± 1.04
	O. faveolata	1.72 ± 0.21	1.01 ± 0.1	1.96 ± 0.2	1.25 ± 0.28	0.94 ± 0.04		1.72 ± 0.5
	M. cavernosa	2.04 ± 0.22^{a}	1.55 ± 0.13^{a}	1.49 ± 0.15^{a}	0.63 ± 0.11^{b}	1.65 ± 0.11^{a}		0.52 ± 0.11^{b}
	P. strigosa	3.69 ± 0.48^{a}	2.2 ± 0.47$^{a/b}$	1.63 ± 0.42^{b}	1.34 ± 0.32^{b}	1.54 ± 0.15^{b}		1.06 ± 0.51^{b}
P_M (μmol O_2 protein^{-1} h^{-1})	*O. annularis*	1.27 ± 0.07^{a}	1.2 ± 0.1^{a}	1.04 ± 0.25^{a}	0.27 ± 0.12^{b}	0.92 ± 0.08^{a}		0.16 ± 0.08^{b}
	O. faveolata	0.95 ± 0.17^{a}	0.48 ± 0.07$^{a/b}$	1.1 ± 0.16^{a}	0.3 ± 0.13^{b}	0.68 ± 0.11$^{a/b}$		0.13 ± 0.004^{b}
	M. cavernosa	0.32 ± 0.02^{a}	0.41 ± 0.09^{a}	0.32 ± 0.04^{a}	0.06 ± 0.006^{b}	0.34 ± 0.01^{a}		0.02 ± 0.01^{b}
	P. strigosa	0.83 ± 0.12^{a}	0.51 ± 0.03$^{a/b/c}$	0.58 ± 0.05$^{a/b}$	0.2 ± 0.04$^{b/c}$	0.68 ± 0.12^{a}		0.05 ± 0.02^{c}

Manuscript: Seasonal variation modulates coral sensibility to thermal-stress and explains annual changes in coral productivity. Tim Scheufen, Wiebke E. Krämer, Roberto Iglesias-Prieto, and Susana Enríquez

Table S10: *Two-way ANOVA for the analysis of the Fv/Fm variation along the experiment*

		March				October			
		Sum Sq	Df	F value	P	Sum Sq	Df	F value	P
O. annularis	Intercept	0.109	1	41.752	**<0.01**	0.174	1	54.632	**<0.01**
	Day	0.145	1	55.73	**<0.01**	0.056	1	17.569	**<0.01**
	Temperature	0.002	1	0.704	0.403	0.014	1	4.356	**<0.05**
	Day x Temperature	0.0175	1	67.183	**<0.01**	0.062	1	19.573	**<0.01**
	Residuals	0.381	146			0.554	174		
O. faveolata	Intercept	0.099	1	37.64	**<0.01**	0.2	1	48.506	**<0.01**
	Day	0.111	1	42.108	**<0.01**	0.104	1	25.182	**<0.01**
	Temperature	0.0001	1	0.052	0.82	0.011	1	2.672	0.104
	Day x Temperature	0.131	1	49.634	**<0.01**	0.119	1	28.868	**<0.01**
	Residuals	0.383	145			0.882	214		
M. cavernosa	Intercept	0.123	1	94.076	**<0.01**	0.089	1	29.176	**<0.01**
	Day	0.19	1	145.386	**<0.01**	0.829	1	272.657	**<0.01**
	Temperature	0.002	1	1.477	0.226	0.003	1	0.898	0.344
	Day x Temperature	0.226	1	172.219	**<0.01**	0.938	1	308.746	**<0.01**
	Residuals	0.191	146			0.69	227		
P. strigosa	Intercept	0.102	1	743.094	**<0.01**	0.079	1	56.957	**<0.01**
	Day	0.033	1	243.96	**<0.01**	0.101	1	73.272	**<0.01**
	Temperature	0.0004	1	2.98	0.086	0.003	1	1.87	0.174
	Day x Temperature	0.041	1	297.866	**<0.01**	0.114	1	82.097	**<0.01**
	Residuals	0.02	146			0.144	104		

Manuscript: Seasonal variation modulates coral sensibility to thermal-stress and explains annual changes in coral productivity. Tim Scheufen, Wiebke E. Krämer, Roberto Iglesias-Prieto, and Susana Enríquez

Table S11: *Average values ± SE of Fv/Fm for the response to thermal-stress of the winter and summer phenotypes. Different letters indicate differences between temperature treatments within each species (two-way ANOVA, P<0.05, Tukey Post-hoc P<0.05).*

		March				October			
		O. annularis	*O. faveolata*	*M. cavernosa*	*P. strigosa*	*O. annularis*	*O. faveolata*	*M. cavernosa*	*P. strigosa*
Day 0	28ºC	0.61 ± 0.01^{a}	0.64 ± 0.001^{a}	0.66 ± 0.001^{a}	0.64 ± 0.001^{a}	$0.64 \pm 0.01^{a/b/c}$	$0.66 \pm 0.01^{a/b}$	$0.64 \pm 0.001^{a/b}$	$0.62 \pm 0.01^{a/b}$
Day 6	28ºC	$0.58 \pm 0.01^{a/b}$	$0.62 \pm 0.01^{a/b/c}$	0.62 ± 0.01^{b}	0.61 ± 0.001^{b}	$0.63 \pm 0.01^{a/b/c}$	$0.65 \pm 0.01^{a/b/c}$	0.62 ± 0.01^{b}	$0.61 \pm 0.01^{a/b}$
	30ºC	$0.53 \pm 0.01^{b/c}$	$0.6 \pm 0.01^{b/c}$	0.58 ± 0.01^{c}	0.6 ± 0.001^{c}	$0.61 \pm 0.01^{b/c}$	$0.62 \pm 0.001^{b/c}$	0.59 ± 0.01^{b}	$0.58 \pm 0.01^{b/c}$
	32ºC	0.46 ± 0.01^{d}	0.52 ± 0.01^{d}	0.5 ± 0.01^{d}	0.55 ± 0.001^{e}	$0.53 \pm 0.02^{d/e}$	0.54 ± 0.02^{d}	0.52 ± 0.01^{c}	0.51 ± 0.01^{d}
Day 10	28ºC	$0.58 \pm 0.01^{a/b}$	$0.62 \pm 0.01^{a/b/c}$	0.62 ± 0.001^{b}	0.62 ± 0.001^{b}	0.68 ± 0.01^{a}	0.69 ± 0.001^{a}	0.67 ± 0.001^{a}	0.66 ± 0.001^{a}
	30ºC	0.52 ± 0.01^{c}	0.59 ± 0.01^{c}	0.57 ± 0.01^{c}	0.59 ± 0.001^{c}	$0.65 \pm 0.01^{a/b}$	$0.67 \pm 0.01^{a/b}$	$0.63 \pm 0.001^{a/b}$	$0.63 \pm 0.001^{a/b}$
	32ºC	0.4 ± 0.02^{e}	0.47 ± 0.02^{e}	0.42 ± 0.01^{e}	0.53 ± 0.001^{e}	$0.59 \pm 0.02^{c/d}$	$0.59 \pm 0.03^{c/d}$	0.52 ± 0.01^{c}	$0.55 \pm 0.01^{c/d}$
Day 20	28ºC					$0.64 \pm 0.01^{a/b/c}$	$0.65 \pm 0.01^{a/b/c}$	$0.63 \pm 0.001^{a/b}$	$0.61 \pm 0.001^{a/b}$
	30ºC								
	32ºC					0.5 ± 0.02^{e}	0.48 ± 0.02^{e}	0.16 ± 0.02^{d}	0.35 ± 0.01^{e}
